# Supplementary material for: Investigating mental defeat in individuals with chronic pain: Protocol for a longitudinal experience sampling study
Source: BMJ Open. 2023 Feb 6;13(2):e066577. doi: 10.1136/bmjopen-2022-066577 (PMC9906405; doi:10.1136/bmjopen-2022-066577)
Supplement: Supplementary data [file bmjopen-2022-066577supp002.pdf]

Within
Warwick Study of Mental Defeat in Chronic Pain

WARWICK
THE UNIVERSITY OF WARWICK

COVID Checklist

The checklist is administered by a member of the research team over the telephone once per person, per timepoint (T1 and T2). This is to ensure reduced risk of virus transmission and to ensure that the sleep, pain, mood and activity measured within the study are representative of their typical chronic pain and not influenced by COVID infection. The COVID checklist requires participants to answer three questions to determine any recent exposure:

1. Have you experienced any of the main COVID-19 symptoms in the last 48 hours?
2. In the last 7 days have you come into close contact with anyone who has tested positive for COVID-19 to your knowledge?
3. Are you or is anyone in your household/bubble currently self-isolating?

If a participant answers ‘yes’ to one or multiple questions, they will be asked to postpone their participation until they no longer test positive. Data obtained from the checklist will not be analysed as their purposes are for health and safety and to maintain data integrity.
