# Supplementary material for: Investigating mental defeat in individuals with chronic pain: Protocol for a longitudinal experience sampling study
Source: BMJ Open. 2023 Feb 6;13(2):e066577. doi: 10.1136/bmjopen-2022-066577 (PMC9906405; doi:10.1136/bmjopen-2022-066577)
Supplement: Author's manuscript [file bmjopen-2022-066577.draft_revisions.pdf]

BMJ Open is committed to open peer review. As part of this commitment we make the peer review history of every article we publish publicly available.

When an article is published we post the peer reviewers' comments and the authors' responses online. We also post the versions of the paper that were used during peer review. These are the versions that the peer review comments apply to.

The versions of the paper that follow are the versions that were submitted during the peer review process. They are not the versions of record or the final published versions. They should not be cited or distributed as the published version of this manuscript.

BMJ Open is an open access journal and the full, final, typeset and author-corrected version of record of the manuscript is available on our site with no access controls, subscription charges or pay-per-view fees (http://bmjopen.bmj.com).

If you have any questions on BMJ Open's open peer review process please email info.bmjopen@bmj.com

BMJ Open

Investigating Mental Defeat in Patients with Chronic Pain: Protocol for a Longitudinal Experience Sampling Study

Journal:	*BMJ Open*
Manuscript ID	bmjopen-2022-066577
Article Type:	Protocol
Date Submitted by the Author:	12-Jul-2022
Complete List of Authors:	Gillett, Jenna; University of Warwick, Psychology<br>Karadag, Paige; University of Warwick, Psychology<br>Themelis, Kristy; University of Warwick, Psychology<br>Li, Yu-Mei; Bielefeld University, Psychology<br>Lemola, Sakari ; Bielefeld University, Psychology<br>Balasubramanian, Shyam; University Hospitals Coventry and Warwickshire NHS Trust<br>Singh, Swaran; University of Warwick Warwick Medical School, Mental Health and Wellbeing<br>Tang, Nicole; University of Warwick, Psychology
Keywords:	PAIN MANAGEMENT, MENTAL HEALTH, STATISTICS & RESEARCH METHODS
Note: The following files were submitted by the author for peer review, but cannot be converted to PDF. You must view these files (e.g. movies) online.	
actiwatch_video_supplementary material.mp4	

SCHOLARONE™
Manuscripts

TITLE: Investigating Mental Defeat in Patients with Chronic Pain: Protocol for a Longitudinal Experience Sampling Study

AUTHORS: Jenna L Gillett (PhD Researcher)[1]*, Paige Karadag (Research Assistant)[1]*, Kristy Themelis (Post-Doctoral Research Fellow)[1], Yu-Mei Li (Post-Doctoral Research Fellow)[2], Sakari Lemola (Professor)[2], Shyam Balasubramanian (Consultant)[3], Swaran Singh (Professor)[4], Nicole KY Tang (Professor)[1 a]

[1]Department of Psychology, University of Warwick, Coventry, United Kingdom

[2]Department of Psychology, Bielefeld University, Bielefeld, Germany

[3]University Hospital Coventry and Warwickshire NHS Trust, Coventry, United Kingdom

[4]Warwick Medical School, University of Warwick, Coventry, United Kingdom

*Joint first authorship (equal contributions)

[a]Corresponding Author: Nicole KY Tang, Email: n.tang@warwick.ac.uk

ORCID IDs:

J.L. Gillett: 0000-0002-7115-9938

P. Karadag: 0000-0003-2655-7109

K. Themelis: 0000-0002-0022-5272

S. Lemola: 0000-0002-1314-6194

S. Balasubramanian: 0000-0001-5149-3595

N.K.Y. Tang: 0000-0001-7836-9965

For peer review only

ABSTRACT

Introduction: Previous qualitative and cross-sectional research has identified a strong sense of mental defeat in people with chronic pain who also experience the greatest levels of distress and disability. This study will adopt a longitudinal experience sampling design to examine the within-person link between the sense of mental defeat and distress and disability associated with chronic pain.

Methods and Analysis: We aim to recruit 198 participants (aged 18-65 years) with chronic pain, to complete two waves of experience sampling over 1 week, 6 months apart (Time 1 and Time 2). During each wave of experience sampling, the participants are asked to complete 3 short online surveys per day, to provide in-the-moment ratings of mental defeat, pain, physical and social activity, stress, mood, self-compassion, and attention using visual analogue scales (VAS). Sleep and physical activity will be measured using a daily diary as well as with wrist-actigraphy worn continuously by participants throughout each wave. Linear mixed models and Gaussian graphical models will be fit to the data to: (1) examine the within-person, day-to-day association of mental defeat with outcomes (i.e., pain, physical/social activity, medication use and sleep) (2) examine the dynamic temporal and contemporaneous networks of mental defeat with all outcomes and the hypothesised mechanisms of outcomes (i.e., perceived stress, mood, attention, and self-compassion).

For peer review only

STRENGTHS AND LIMITATIONS OF THE STUDY:

- This study provides the first longitudinal investigation of mental defeat in chronic pain to shed light on its temporal links with outcomes.
- A range of outcomes and hypothesised mechanisms will be assessed including pain, physical and social activity, medication use, sleep, stress, mood, attention, and self-compassion. Measures are repeated over a one-week period, at two time-points each six-months apart.
- This study will use both self-reported and objective estimates of sleep and physical activity, via diaries and actigraphy longitudinally.
- The research is done remotely, at the participant's convenience and within their natural environment.
- Considerations must be given to effects of participants' COVID exposure on recruitment, subsequent attrition, and possible findings despite having had appropriate COVID screening and health and safety procedures in place.

For peer review only

1. INTRODUCTION

Chronic pain is characterised as pain that persists or recurs beyond 3 months (1). It is highly prevalent, affecting around 30% of the population worldwide (2–4). Chronic pain conditions, namely low back pain and headaches, are consistently the top causes of years lived with disability (2,5) and reduced quality of life (6). People with chronic pain are three times as likely to have depression and anxiety disorders (7) and twice as likely to present a risk of suicide compared to the general population (8). Whilst some individuals manage to cope with the pain, others struggle to maintain daily activities. Understanding the factors that determine whether an individual can feel and function well- despite persistent pain- is crucial to advancing non-pharmacological management approaches for chronic pain, which have so far had a modest impact (9).

A concept proposed to help explain differences in the experience of pain-related distress and disability is mental defeat; a cognitive construct characterised by negative self-appraisals in relation to pain (10,11). The concept of defeat has its theoretical underpinnings in the study of post-traumatic stress disorder (PTSD) (12–14) and depression (15,16), where it is respectively defined as the perceived loss of autonomy and a natural response to the loss of social status in a conflict situation. Empirical research has shown that a strong sense of mental defeat is associated with severe PTSD symptoms and poorer response to exposure treatment (12,14,17). The perception of defeat has also been shown to predict symptoms of depression independent of hopelessness (15), and has been implicated in psychological models of suicidal behaviour and suicidality (18–20).

Mental defeat in the context of chronic pain encapsulates people's psychological response to perceived threats of one's physical and psychological

For peer review only - http://bmjopen.bmj.com/site/about/guidelines.xhtml

autonomy. Daily experience of living with persistent and debilitating pain which does not respond to treatment is thought to be a repeated trigger of mental defeat, prompting negative appraisals of self in relation to pain (21). Consistently, qualitative explorations of patient experience have revealed patients reporting “defeat of the mind” and “the pain is taking over”, with the pain seen as “an enemy” that “belittles [them] as a person” (11,22,23) .

Using the Pain Self Perception Scale to measure mental defeat, it has been found that treatment-seeking patients with chronic pain have elevated levels of mental defeat compared to: patients with acute pain, patients with anxiety disorders, community volunteers with chronic pain, community volunteers with acute pain and pain-free volunteers (21). Mental defeat has also been found to be the predictor explaining the most variance in pain interference, depression, and psychological disability among chronic pain patients seeking specialist treatment, when compared to pain intensity, health anxiety, worry rumination, and pain catastrophising (11). It has moderate associations with sleep disturbances and functional disability (11) and negatively relates to pain self-efficacy even when anxiety, depression, pain catastrophising, and hopelessness are controlled for (24). Furthermore, mental defeat predicts suicide intent in patients with chronic pain above and beyond pain intensity (25). In pain-free volunteers, an activated sense of mental defeat appears to operate independently from existing pain-related psychological constructs such as pain catastrophising, in influencing mood and attentional disengagement from nociceptive stimuli (26). Together, these findings suggest how a person’s self-perception in relation to pain matters in terms of predicting and explaining outcomes.

However, most of the aforementioned studies of mental defeat in chronic pain are cross-sectional in design, and more direct evidence is required to establish the

For peer review only

temporal and casual association implicated. This study adopts the experience sampling methodology (ESM) involving *in vivo* data gathering (using actigraphy, sleep diaries and daily online surveys) to examine the day-to-day association between mental defeat, symptoms, distress, and disability associated with chronic pain. Data will be collected over 7 days, allowing the study of any temporal within-person relationships which may provide insight into clinically relevant questions such as whether mental defeat will be followed by higher pain, increased stress, greater attention to pain, increased medication usage, reduced physical and social activity and poorer sleep. The experience sampling exercise is repeated at 6 months to investigate how these indices may change over time and translate into distress and disability long-term. The primary objectives of this study are: (1) examine the within-person, day-to-day association of mental defeat with outcomes (i.e., pain, physical/social activity, medication use and sleep), and (2) examine the dynamic temporal and contemporaneous networks of mental defeat with all outcomes and the hypothesised mechanisms of outcomes (i.e., perceived stress, mood, attention, and self-compassion).

Based on previous work (e.g. 11,21,25) we hypothesise that, for an individual, a strong sense of mental defeat will be associated with subsequent greater reports of pain, reduced physical and social activity, possible increased use of medication, and poorer sleep. Examinations of the dynamic temporal and contemporaneous networks of mental defeat with mechanisms and outcomes are novel and exploratory.

2. METHOD

2.1 Study Design

For peer review only - http://bmjopen.bmj.com/site/about/guidelines.xhtml

As depicted in Figure 1, ESM are used to gather data prospectively over 1-week; at two-time points, each six months apart (T1 and T2). The length and frequency of assessment reflect our attempt to balance information needs with concerns of participation burden and possible attrition, participants are asked to continuously wear a medical-grade actigraphy device (MotionWatch 8©, manufactured by CamNTech Ltd) for 8 nights/7 days. They are asked to complete a sleep diary and respond to 3 short online surveys during the day. These surveys will provide in-the-moment measures of mental defeat, pain, medication use, physical activity and social activity, stress, mood, attention, and self-compassion using visual analogue scales (VAS).

Insert Figure 1 about here

2.2 Participants

Participants are adults aged 18-65 living with chronic non-cancer pain that has been present or recurring for more than 3 months (27,28). Participant inclusion and exclusion criteria are outlined in Table 1.

Table 1.
PARTICIPANT INCLUSION & EXCLUSION CRITERIA FOR PARTICIPATION

Inclusion Criteria:
➢ Aged between 18 – 65 (for focussing our study on working-age population).<br>➢ Experience chronic non-cancer pain for at least 3 months.<br>➢ Stable treatment for duration of the study (6 months).<br>➢ English-speaking (for understanding and implementing the data collection procedure).<br>➢ Living in the UK (for postage of equipment).<br>➢ Be able to provide informed consent.
Exclusion Criteria:

For peer review only

- Have any significant comorbid psychiatric (e.g., psychosis), medical (e.g., coronary heart diseases), neurological (e.g., Alzheimer's, Parkinson's, Epilepsy) or life-threatening conditions (e.g., cancer) that would impact pain experience, impede the ability to provide informed consent or complete the study.
- Have any other significant comorbid sleep disorder, e.g., sleep apnoea, restless leg syndrome, periodic limb movement disorder, narcolepsy or circadian rhythm disorders, which in the opinion of the research team would cofound the results of the study.
- Have elective surgery or procedures requiring general anaesthetic during the study.
- Have participated in another research study using an investigational product in the past 3-months.

We aim to recruit 198 participants to factor in an anticipated 20% attrition at T2. This will give 158 participants who complete the experience sampling procedure at both timepoints to generate up to 6636 temporally structured ratings for analysis (3 ratings x 7 days x 2 timepoints x 158 participants). There will be a maximum of 2528 observations of sleep data (8 nights of actigraphic data x 2 timepoints x 158 participants) and 7 days' of physical activity count data at 30-second epoch. This will give sufficient power to perform the planned analyses using Multilevel Mixed-Effects Models (29) and Graphical Gaussian Models (30).

2.3 Recruitment

Recruitment of participants started in April 2021 and is expected to end in May 2023. A variety of recruitment methods are being used, including social media, the NIHR Clinical Research Network, public engagement events, and peer-led support groups. We are also using online recruitment platforms to capture individuals with chronic pain with registered interest to take part in research. Finally, chronic pain patients at University Hospitals Coventry and Warwickshire are given information about the study during pain clinic appointments.

For peer review only

2.4 Measures

2.4.1 Screening Questionnaire

A brief online screening questionnaire is administered to assess eligibility. To provide the relevant information against the *a priori* inclusion and exclusion criteria, the screening questionnaire determines basic demographics (e.g., age, sex, ethnicity, employment status, education level) and health indicators (e.g., body mass index, average alcohol intake, smoking status). It also checks for pain characteristics, current treatment plans, e.g. plans for surgery in the next 6-months and current participation in clinical trials. Lastly, it considers comorbid health conditions, including the presence of any psychiatric, medical, neurological or sleep disorders.

2.4.2 Baseline Questionnaire

Eligible participants are asked to complete a baseline questionnaire which includes validated measures of variables related to mental defeat, pain, physical and social activity, sleep, psychological states and quality of life (see Table 2 for full list). Data from these questionnaires are not used in the planned analyses of the current study except for characterising the sample at baseline.

For peer review only

Table 2.
QUESTIONNAIRE MEASURES INCLUDED

Measure	Scale Used
**Key Variable of Interest**	
Mental defeat	*Pain Self-Perception Scale (PSPS)*(21)
**Pain-Related Measures**	
Pain intensity & interference	*Brief Pain Inventory-Short Form (BPI-sf)*(31)
Pain vigilance and awareness	*Pain Vigilance & Awareness Questionnaire (PVAQ)*(32)
Pain-related fear of movement	*Tampa Scale of Kinesiophobia (TSK-11)*(33)

Patterns of activity (pain-specific)	*Patterns of Activity Measure for Pain (POAM-P)*(34)
Pain catastrophising	*Pain Catastrophizing Scale (PCS)*(35)
Pain self-efficacy	*Pain Self-Efficacy Questionnaire (PSEQ)*(36)
**Physical & Social Activity Measures**	
Physical activity	*International Physical Activity Questionnaire (IPAQ)*(37)
Social activity	*Social Activity Log (SAL)*(38)
**Sleep-Related Measure**	
Insomnia symptom severity	*Insomnia Severity Index (ISI)*(39)
**Psychological States**	
Stress	*Perceived Stress Scale (PSS)*(40)
Anxiety & depression	*Hospital Anxiety & Depression Scale (HADS)*(41)
Suicidal behaviour	*Suicidal Behaviour Questionnaire Revised (SBQ-R)*(42)
Self-compassion	*Self-Compassion Scale Short Form (SCS-sf)*(43)
**Quality of Life Measure**	
General health and quality of life	*EQ-5D-5L*(44)

2.4.3 Sleep Diary

The morning section of the Consensus Sleep Diary (45) is administered daily throughout the tracking period to collect self-reported information on sleep. The sleep diary asks participants what time they went to bed, what time they attempted to go to sleep, how long it took them to fall asleep (sleep onset latency), how many times did they wake up from sleep (not including final awakening), final wake up time, what time did they get out of bed, total sleep duration (hours and minutes), perceived sleep-quality and how rested or refreshed they felt after waking. Participants can also provide additional information that they feel is relevant to their sleep. We added two extra questions to the sleep diary to obtain in-the-moment ratings of pain and mood upon waking. These possible covariates are assessed via

For peer review only

two VAS both ranging from 0-10, whereby for pain 0= *no pain at all* and 10= *worst pain imaginable* and for mood 0= *very bad* and 10= *very good*.

2.4.4 Daily Survey

The daily survey allows participants to provide ratings of mental defeat, pain, medication use, physical and social activity, stress, mood, attention, and self-compassion (see Figure 1 for a schedule of administration). We use adapted or shortened versions of original scales to decrease participant burden. The surveys are short (<5 minutes completion time) and are equally spaced 2.5 hours apart to capture experiences at different time points throughout the day. The surveys are sent out following a choice of pre-determined schedules to match participants' typical sleep-wake patterns. The earliest schedule starts with a sleep diary at 06:30 and the first daily survey commences at 09:00, whereas the latest schedule starts with a sleep diary at 13:30 and the first daily survey commences at 16:00. The timing of these measures avoids unsociable hours, as no prompts arrive between the hours of 23:00 and 06:00 inclusive.

We use Survey Signal to send out auto-prompts at specified times via SMS to participants' smartphones. This enables accessibility for participants to complete surveys in a timely fashion, while remaining convenient, and does not require an app download or adjusted personal mobile settings. The surveys are administered, recorded, and returned via Qualtrics and are time-stamped at the time of commencement and completion. The daily surveys comprise multiple VAS with varying left to right anchors, as shown in Table 3.

Table 3.
DAILY SURVEY RATING SCALES

Construct	Item (measure)	Scale	Anchors

For peer review only

Mental defeat	Since waking up today, how much has the pain brought back to life memories of times when you felt the pain had taken over?	0 – 10	0= not at all to 10= very much so
Pain intensity	What is your current pain level?	0 – 10	0 = no pain to 10 = worst pain imaginable
Pain interference	Since waking up today, how much has your pain impacted on…<br>a) Your daily routine (including work)?<br>b) Your relationship(s)?<br>c) How you think or feel about the future?<br>d) How you think or feel about yourself?	0 – 10	0= not much impact/interference to 10 = a great deal of impact/interference
Medication use	Since waking up today, would you say that you have taken more or less medication than usual?	-5 – 5	-5= a lot less than usual, 0 = no difference, 5 = a lot more than usual
Physical activity	Since waking up today, how physically active has you been?	0 – 10	0= not physically active at all to 10= very physically active
Social activity	Since waking up today, how socially engaged have you been?	0 – 10	0= not socially engaged at all to 10= very socially engaged
Stress	What is your current stress level?	0 – 10	0= no stress at all to 10= highest level of stress possible
Mood	What is your current mood?	0 – 10	0= very bad to 10 = very good
Attention to pain	Since waking up today, how much of the time have you been thinking about your pain?	0 – 10	0= none of the time to 10= a great deal of the time
Focus of attention	Since waking up today, has the focus of your attention been…<br>a) Inward or outward	0 – 10	0 = inward to 10 outward<br>0 = body to 10= mind

For peer review only

	b) On the body or mind		
Self-compassion	Since waking up today, how kind…<br>a) to yourself have you been?<br>b) to others have you been?	0 – 10	0= not at all to 10= very much so

Note. VAS questions with corresponding anchors that are presented in Survey 1 are summarised above. These questions appear identically in Surveys 2 and 3, except instead of starting questions with "since waking up today…" in Surveys 2 and 3 each question begins "in the last 2.5 hours…".

For peer review only

2.4.5 Actigraphy

Actigraphs are light, compact accelerometer-based devices that have been used to generate objective estimates of sleep parameters for several decades (46). Actigraphy has been well-evidenced as a suitable methodology for non-intrusive at-home sleep assessment (47–49). Although polysomnography continues to be considered the gold standard for sleep recording, wrist-actigraphy has the advantage of offering cost-effective continuous recording in participants' home environment (50). Thus providing more ecologically valid information compared to polysomnography (50).

For the present study, MotionWatch 8© actigraphs are worn by participants on their non-dominant wrist during the study. The MotionWatch is a medical-grade tri-axial actigraphy device containing a piezoelectric accelerometer to record duration, integration, and number of movements in all directions. This data enables the research team to chart sleep and physical activity across the experience sampling periods that are then downloaded for analysis using MotionWare© software (Cambridge Neurotechnology Ltd., Cambridge, UK) with validated algorithms. The key sleep parameters we are interested in are sleep efficiency (SE) and total sleep time (TST). The key physical activity parameter is total activity counts tabulated by week, day, and/or hour.

2.5 Procedure

To participate, individuals respond to a study advert via phone, email or by following a direct link to the information leaflet. Interested participants are required to complete the screening questionnaire and contact information form, following which a member of the research team determines eligibility to the study by checking against the inclusion/exclusion criteria. Individuals who meet the inclusion criteria are informed of their eligibility via email and invited to participate. Individuals who do not meet the inclusion criteria are informed via email that they are not eligible, thanked and debriefed.

Eligible participants that agree to participate are sent a link to an online consent form to complete via Qualtrics. Once informed consent has been obtained, participants have a phone call with a member of the research team to arrange their participation and highlight some key training points for the study. All participants receive the study materials (an invitation letter, a cleaned and packaged MotionWatch for borrowed use, two individually wrapped disinfectant wipes, an addressed, pre-paid return envelope, and an information booklet) via UK postal delivery. To accompany the information booklet, participants are emailed a link to an instructional video (see supplementary file 1) demonstrating how to use the MotionWatch devices appropriately and reiterating the schedule of measurement/engagement required during participation. Participants are instructed to wear the MotionWatch continuously for the 7-day experience sampling period and are required to press an event marker on the device when they plan to go to sleep and when they get out of bed, following their main sleep period. Participants are also asked to answer the sleep diary and three short surveys each day.

For peer review only

The timing of the daily surveys/sleep diary are individually anchored by participants' typical sleep-wake patterns, to accommodate and control for variations in individuals' circadian rhythms. For example, if a participant indicates that they usually wake at 08:00, a typical day during the experience sampling period on this schedule would be as follows: upon getting out of bed, the participant presses the event marker on the MotionWatch and will receive the first text message (with a link to the sleep diary) at 08:30 – allowing them to report their sleep experiences as soon as practical after waking. Throughout the day, the participant will receive three further text message prompts, each containing a link to the short online survey. Survey 1 (S1) is received at 11:00, survey 2 (S2) at 13:30 and survey 3 (S3) at 15:00. The links to each survey remains open for 2.5 hours, before expiring at the time the following survey is triggered. Finally, the participant will be required to press the event marker at "lights out" or when beginning trying to sleep. This process is identical for each day in the experience sampling period. After the final awakening on the last day, the participant removes the MotionWatch, and packages it in the box ready for return-postage to the Lab. The MotionWatch data is processed on-site at the Lab, before being formatted into a personalised breakdown for the participant, which is emailed to them along with a gift voucher within two weeks of receipt of the returned equipment. This procedure is repeated in its entirety for the follow-up T2 assessment. The debrief is administered upon study completion.

2.6 Participant Reimbursement

To thank the participants for their time and participation, they are given a £10 gift voucher for each timepoint they complete. Additionally, participants are provided with

For peer review only

a personalised breakdown of their actigraphy data created by the research team. No evaluative feedback on sleep quality and physical activity patterns is given.

2.7 Adverse Event Recording and Management

This is a low-risk observational study, and no major adverse events are anticipated. We offer health and safety training at the outset, instructing participants not to respond to survey text messages if it is not safe to do so, e.g., when driving, operating machinery, or crossing the road. Before participation commences, participants are instructed to report any adverse events that occur during the assessment periods to the research team.

Adverse events that are related to the study and/or unrelated adverse events are recorded and reported to the study sponsor according to reporting requirements. Unrelated and expected adverse events may include but are not limited to illness, hospitalisation or day-surgery that occurs during the assessment period. An adverse event that a small number of participants may experience is discomfort or irritation caused by wearing the MotionWatch. Before participants begin the study, we advise them to inform the researcher if they experience any skin irritation. If the irritation is very mild and they wish to continue, we recommend placing a small piece of tissue underneath the watch or to place the silicon strap on top of their sleeve to avoid direct skin contact. In the unlikely event of an unexpected adverse event that is deemed to be severe and related to the study, the research team would immediately pause the study and send an expedited report to the study sponsor. Events will be followed up until they are resolved or when a final outcome has been reached.

2.8 Patient and Public Involvement

For peer review only

This protocol has been developed in partnership with two patient representatives with lived experience of chronic pain. One representative (PR) provided feedback on participant-facing materials and piloted the MotionWatch for at-home use. DD participated in study-related procedures and also commented on the manuscript for readability.

2.9 COVID Related Considerations

To enable the study to take place during the COVID-19 pandemic, while adhering to governmental and institutional COVID-19 guidance, the operational aspects of the protocol have been adapted for remote participation. The MotionWatch devices are all sanitised, prepared and packaged for participation in a lab environment. A short COVID checklist has been implemented before each wave of tracking (see supplementary file 2).

3. DATA MANAGEMENT PLAN

The study data collected from the questionnaire, sleep diary and daily surveys will be stored securely on Qualtrics and subsequently downloaded as password protected databases to undergo data quality checks and pseudo-anonymisation by the research team. Access to these databases will be restricted to approved members of the research team. Once data completeness and quality are verified, electronic data on Qualtrics will be deleted.

Actigraphic data collected using MotionWatch 8© are downloaded using Motionware© software upon each watch's return to the lab. The downloaded data are saved via their assigned ID number and will be held securely and separately from the study data.

For peer review only - http://bmjopen.bmj.com/site/about/guidelines.xhtml

The Chief Investigator (NT) of the project will act as the data controller. All data generated by the research programme will be analysed by the research team either on site at the University of Warwick or in a private workspace in the event of remote-working, as per the University's Off-Campus Working Policy. In line with the University's Research Code of Conduct, data will be retained in electronic format for at least 10 years from the date of any publication that is based upon it.

4. STATISTICAL METHODS

All participants who meet eligibility criteria will be included in analyses; including those who wish to withdraw from the study but consent to having any data already collected analysed. Those who withdraw and do not consent to the data being analysed will be excluded from analyses.

Descriptive statistics will be used to characterise the sample based on information from the screening and baseline questionnaires. Means and standard deviations/95% confidence intervals will be reported for continuous variables, whereas frequencies and percentages will be used for reporting categorical variables.

To evaluate the within-person temporal relationship between mental defeat (predictor) and pain, physical and social activity, medication use and sleep (outcomes), we will pool the daily survey ratings from all participants across waves of assessment. Linear mixed models with a time-lagged design will be fit to the data. We will fit one model for each outcome. For each analysis, we will first estimate a maximal random effects model with all random slopes and random intercepts (for 'time of day [survey 1, 2, 3]', 'day [1, 2, 3, 4, 5, 6, 7]' and 'wave [1, 2]') and successively simplify the random effect structure until the model converges. Nested

For peer review only

comparisons will be made between the final models with the intercepts-only models. The significance of each model will be assessed using a likelihood ratio test (LRT). P-values will be adjusted using false discovery rate to account for alpha error accumulation (FDR; (51)). The best fitting model will be determined using Aikaike Information Criteria (AIC) (52,53) and Bayesian Information Criteria (BIC) (54), where lower values of the AIC and BIC indicate a better fit. As secondary analyses, we will repeat the above analyses with the hypothesised mechanisms of outcomes (stress, mood, attention, self-compassion) as the dependent variables. Both the statistical software SPSS (IBM Corp., Armonk, NY, USA) and the “lme4” package (55) for R (56) will be used.

To explore the within-individual temporal and contemporaneous relationships as well as between-individual relationships between mental defeat and the outcomes and hypothesised mechanisms of interest, we will use Gaussian graphical models (GGM; (30)). To obtain sufficient power for the GGMs, it is recommended to have more than 20 measurements and at least 20 pairs of comparisons (57). The design of the current study will yield 21 measurements per participant per wave. That will give 20 pairs of comparisons for modelling changes across days, 14 pairs of comparisons for modelling changes within day, and 7 pairs of comparisons for modelling changes at different times of the day. We will therefore not seek to model changes at different times of the day, but to focus on modelling changes across and within day by pooling together data from both waves of assessment.

We will use the mlVAR (Version 0.3.2) package (57,58), or similarly suitable but more up-to-date packages, for R (56) to analyse the multivariate time series data. We will report three kinds of network; temporal (to indicate whether a variable predicts another variable (or itself) at the next measurement point, controlling for all

For peer review only

other variables in the network at the previous measurement point), contemporaneous (to indicate the within-person relationships between variables, having adjusted for the effect of all variables in the network and the temporal effects) and between-person (to indicate relationships between person-means of variables, partialling out the effect of all other variables in the network).

Additional secondary analyses will be performed for research questions other than the 2 main ones stated here. Any deviation(s) from the original statistical plan will be described and justified in our subsequent reportings.

5. ETHICS & DISSEMINATION

This research forms part of the wider MRC-funded Warwick Study of Mental Defeat in Chronic Pain ("WITHIN" Study). The current protocol has been approved by the Health Research Authority and West Midlands – Solihull Research Ethics Committee (Reference Number: 17/WM0053, p, IRAS project ID: 223190). The University of Warwick (Research Impact Services, University of Warwick, Coventry, CV4 7AL) acts as the Sponsor for the study. The study is being conducted in adherence with the Declaration of Helsinki, Warwick Standard Operating Procedures and applicable UK legislation. Results from this study will be written as reports, to be disseminated in peer-reviewed journals, at conferences and patient and public engagement events.

6. AUTHOR'S CONTRIBUTIONS AND ACKNOWLEDGEMENTS

6.1 Authors' Contributions

NT conceived the research idea and developed the theory and plan for this study. JG and PK are joint first author as they contributed equally to all aspects of the study

For peer review only

and are responsible for implementing the protocol, creating study materials, data acquisition and management and drafting the original manuscript. KT and NT are responsible for critical revisions of the manuscript. All authors (JG, PK, KT, Y-ML, SL, SB, SS, NT) contributed to the study development and reviewed, commented, and approved the manuscript.

6.2 Acknowledgements

The authors would like to thank all the participants who are involved in the study and the UK chronic pain charities who have shared our study on relevant webpages and social media platforms. We would also like to thank our PPI representatives (PR & DD) for their input into the study design and feasibility, including Debra Dulake (DD) who also commented on the manuscript. Finally, we would like to thank Michelle Pun, Emily Ashton and Chloe Osei-Cobbana who have supported the data collection process under the Psychology Research Skills Development Scheme (PRSDS) at the University of Warwick.

7. COMPETING INTERESTS

The authors have no competing interests.

8. FUNDING

This protocol is part of a wider study (Warwick Study of Mental Defeat in Chronic Pain that is supported by Medical Research Council (MRC) grant number: MR/S026185/1 to NKYT. For the purpose of open access, the corresponding author has applied a Creative Commons Attribution (CC BY) licence (where permitted by UKRI, 'Open Government Licence' or 'Creative Commons Attribution No-derivatives

For peer review only

(CC BY-ND) licence' may be stated instead) to any Author Accepted Manuscript version arising from this submission. JLG was funded by the University of Warwick Departmental PhD Fellowship, under the supervision of NKYT. The funders have no role in study design, data collection and analysis, decision to publish or preparation of this manuscript.

9. DATA SHARING STATEMENT

Once available, cleaned, anonymised data generated from this study will be made available on open-access repository.

10. REFERENCES

1. Treede RD, Rief W, Barke A, Aziz Q, Bennett MI, Benoliel R, et al. Chronic pain as a symptom or a disease: The IASP Classification of Chronic Pain for the International Classification of Diseases (ICD-11) [Internet]. Vol. 160, Pain. Lippincott Williams and Wilkins; 2019 [cited 2022 Jun 14]. p. 19–27. Available from: https://journals.lww.com/pain/Fulltext/2019/01000/Chronic_pain_as_a_symptom_or_a_disease__the_IASP.3.aspx
2. James SL, Abate D, Abate KH, Abay SM, Abbafati C, Abbasi N, et al. Global, regional, and national incidence, prevalence, and years lived with disability for 354 diseases and injuries for 195 countries and territories, 1990–2017: a systematic analysis for the Global Burden of Disease Study 2017. Lancet. 2018 Nov 10;392(10159):1789–858.
3. Dahlhamer J, Lucas J, Zelaya, C, Nahin R, Mackey S, DeBar L, et al. Prevalence of Chronic Pain and High-Impact Chronic Pain Among Adults —

For peer review only

United States, 2016. Morb Mortal Wkly Rep [Internet]. 2018 Sep 9 [cited 2022 Jun 14];67(36):1001–6. Available from: /pmc/articles/PMC6146950/

4. Cohen SP, Vase L, Hooten WM. Chronic pain: an update on burden, best practices, and new advances [Internet]. Vol. 397, The Lancet. Elsevier; 2021 [cited 2022 Jun 14]. p. 2082–97. Available from: http://www.thelancet.com/article/S0140673621003937/fulltext

5. Vos T, Abajobir AA, Abbafati C, Abbas KM, Abate KH, Abd-Allah F, et al. Global, regional, and national incidence, prevalence, and years lived with disability for 328 diseases and injuries for 195 countries, 1990-2016: A systematic analysis for the Global Burden of Disease Study 2016. Lancet [Internet]. 2017 Sep 16 [cited 2022 Jun 29];390(10100):1211–59. Available from: https://research.polyu.edu.hk/en/publications/global-regional-and-national-incidence-prevalence-and-years-lived

6. Fernández A, Saameño JÁB, Pinto-Meza A, Luciano JV, Autonell J, Palao D, et al. Burden of chronic physical conditions and mental disorders in primary care. Br J Psychiatry [Internet]. 2010 Apr [cited 2022 Jun 14];196(4):302–9. Available from: https://www.cambridge.org/core/journals/the-british-journal-of-psychiatry/article/burden-of-chronic-physical-conditions-and-mental-disorders-in-primary-care/1F431966FA03440CA86EE7B2B2028FB6

7. Von Korff M, Crane P, Lane M, Miglioretti DL, Simon G, Saunders K, et al. Chronic spinal pain and physical-mental comorbidity in the United States: Results from the national comorbidity survey replication. Pain [Internet]. 2005 Feb [cited 2022 Jun 28];113(3):331–9. Available from: https://journals.lww.com/pain/Fulltext/2005/02000/Chronic_spinal_pain_and_physical_mental.13.aspx

For peer review only

8. Tang NKY, Crane C. Suicidality in chronic pain: A review of the prevalence, risk factors and psychological links [Internet]. Vol. 36, Psychological Medicine. Cambridge University Press; 2006 [cited 2022 Jun 14]. p. 575–86. Available from: https://www.cambridge.org/core/journals/psychological-medicine/article/abs/suicidality-in-chronic-pain-a-review-of-the-prevalence-risk-factors-and-psychological-links/D3093AA52A611348B8F90CF0972380C5

9. Williams AC d. C, Fisher E, Hearn L, Eccleston C. Psychological therapies for the management of chronic pain (excluding headache) in adults [Internet]. Vol. 2020, Cochrane Database of Systematic Reviews. John Wiley and Sons Ltd; 2020 [cited 2022 Jun 14]. Available from: https://www.cochranelibrary.com/cdsr/doi/10.1002/14651858.CD007407.pub4/full

10. Tang NKY, Salkovskis PM, Hanna M. Mental Defeat in Chronic Pain: Initial Exploration of the Concept. Clin J Pain [Internet]. 2007 Mar [cited 2020 Apr 16];23(3):222–32. Available from: http://journals.lww.com/00002508-200703000-00005

11. Tang NKY, Goodchild CE, Hester J, Salkovskis PM. Mental defeat is linked to interference, distress and disability in chronic pain. Pain. 2010 Jun 1;149(3):547–54.

12. Ehlers A, Maercker A, Boos A. Posttraumatic stress disorder following political imprisonment: The role of mental defeat, alienation, and perceived permanent change. J Abnorm Psychol [Internet]. 2000 [cited 2022 Jun 28];109(1):45–55. Available from: /record/2000-13847-006

13. Dunmore E, Clark DM, Ehlers A. A prospective investigation of the role of cognitive factors in persistent Posttraumatic Stress Disorder ({PTSD}) after

For peer review only

physical or sexual assault. Behav Res Ther. 2001 Sep;39(9):1063–84.

14. Dunmore E, Clark DM, Ehlers A. Cognitive factors involved in the onset and maintenance of posttraumatic stress disorder (PTSD) after physical or sexual assault. Behav Res Ther. 1999 Sep 1;37(9):809–29.

15. Gilbert P, Allan S. The role of defeat and entrapment (arrested flight) in depression: An exploration of an evolutionary view. Psychol Med [Internet]. 1998 [cited 2022 Jun 14];28(3):585–98. Available from: https://www.cambridge.org/core/journals/psychological-medicine/article/abs/role-of-defeat-and-entrapment-arrested-flight-in-depression-an-exploration-of-an-evolutionary-view/A28C75514B33418F797269B1C276AC87

16. Price J, Sloman L, Gardner R, Gilbert P, Rohde P. The social competition hypothesis of depression [Internet]. Vol. 164, British Journal of Psychiatry. Cambridge University Press; 1994 [cited 2022 Jun 14]. p. 309–15. Available from: https://www.cambridge.org/core/journals/the-british-journal-of-psychiatry/article/abs/social-competition-hypothesis-of-depression/18622D076771AB200A26D318AF52A1D9

17. Ehlers A, Clark DM, Dunmore E, Jaycox L, Meadows E, Foa EB. Predicting response to exposure treatment in PTSD: The role of mental defeat and alienation. J Trauma Stress [Internet]. 1998 Jul 1 [cited 2022 Jun 28];11(3):457–71. Available from: https://onlinelibrary.wiley.com/doi/full/10.1023/A%3A1024448511504

18. Williams M. Cry of Pain: Understanding Suicide and the Suicidal Mind [Internet]. 2014 [cited 2022 Jun 14]. 304 p. Available from: https://books.google.co.uk/books?hl=en&lr=&id=msamAgAAQBAJ&oi=fnd&pg

For peer review only

=PT6&dq=+Williams,+M.+(2014).+Cry+of+pain:+understanding+suicide+and+the+suicidal+mind.+Hachette+UK.&ots=ssXWHmmFa7&sig=k3HIS7rgmKO0CXiZiu5yLb4FkBA&redir_esc=y#v=onepage&q=Williams%2C M.

19. O'Connor RC, Kirtley OJ. The integrated motivational-volitional model of suicidal behaviour [Internet]. Vol. 373, Philosophical Transactions of the Royal Society B: Biological Sciences. The Royal Society; 2018 [cited 2022 Jun 14]. Available from: https://royalsocietypublishing.org/doi/10.1098/rstb.2017.0268

20. Johnson J, Gooding PA, Wood AM, Tarrier N. Resilience as positive coping appraisals: Testing the schematic appraisals model of suicide (SAMS). Behav Res Ther [Internet]. 2010 Mar 1 [cited 2020 Mar 25];48(3):179–86. Available from: https://reader.elsevier.com/reader/sd/pii/S0005796709002484?token=047BFC4DC2AA4BAC774ED9741F8C3365F5796A2CDB6555ABD4E520173D205170BFF3942B9E193578519667E13A37C052&originRegion=eu-west-1&originCreation=20211006105621

21. Tang NKY, Salkovskis PM, Hanna M. Mental defeat in chronic pain: Initial exploration of the concept. Clin J Pain [Internet]. 2007 Mar [cited 2022 Jun 14];23(3):222–32. Available from: https://journals.lww.com/clinicalpain/Fulltext/2007/03000/Mental_Defeat_in_Chronic_Pain__Initial_Exploration.5.aspx

22. Smith JA, Osborn M. Pain as an assault on the self: An interpretative phenomenological analysis of the psychological impact of chronic benign low back pain. Psychol Heal [Internet]. 2007 Jul [cited 2022 Jun 14];22(5):517–34. Available from: https://www.tandfonline.com/doi/abs/10.1080/14768320600941756

For peer review only

23. Tang NKY, Salkovskis PM, Hodges A, Soong E, Hanna MH, Hester J. Chronic pain syndrome associated with health anxiety: A qualitative thematic comparison between pain patients with high and low health anxiety. Br J Clin Psychol [Internet]. 2009 Mar 1 [cited 2022 Jun 14];48(1):1–20. Available from: https://onlinelibrary.wiley.com/doi/full/10.1348/014466508X336167

24. Hazeldine-Baker CE, Salkovskis PM, Osborn M, Gauntlett-Gilbert J. Understanding the link between feelings of mental defeat, self-efficacy and the experience of chronic pain. Br J Pain [Internet]. 2018 May 16 [cited 2022 Jun 14];12(2):87–94. Available from: http://www.ncbi.nlm.nih.gov/pubmed/29796260

25. Tang NKY, Beckwith P, Ashworth P. Mental defeat is associated with suicide intent in patients with chronic pain. Clin J Pain. 2016;32(5):411–9.

26. Collard VEJ, Gillett JL, Themelis K, Tang NKY. An exploratory investigation into the effects of mental defeat on pain threshold, pain rating, pain anticipation, and mood. Curr Psychol [Internet]. 2021 Mar 10 [cited 2022 Jun 14];1–12. Available from: https://link.springer.com/article/10.1007/s12144-021-01548-3

27. Raja SN, Carr DB, Cohen M, Finnerup NB, Flor H, Gibson S, et al. The revised International Association for the Study of Pain definition of pain: concepts, challenges, and compromises [Internet]. Vol. 161, Pain. NIH Public Access; 2020 [cited 2022 Jun 14]. p. 1976–82. Available from: /pmc/articles/PMC7680716/

28. NICE. Chronic pain (primary and secondary) in over 16s: assessment of all chronic pain and management of chronic primary pain [Internet]. NICE Guidelines. 2021 [cited 2022 Jun 14]. NICE guideline [NG193]. Available from:

For peer review only

https://www.ncbi.nlm.nih.gov/books/NBK569983/bin/niceng193er6_bm1.pdf

29. Magezi D a. Linear mixed-effects models for within-participant psychology experiments: an introductory tutorial and free, graphical user interface (LMMgui). Front Psychol [Internet]. 2015;6(January):1–7. Available from: http://journal.frontiersin.org/Journal/10.3389/fpsyg.2015.00002/abstract

30. Epskamp S, Borsboom D, Fried EI. Estimating psychological networks and their accuracy: A tutorial paper. Behav Res Methods [Internet]. 2018 Feb 1 [cited 2022 Jun 14];50(1):195–212. Available from: https://link.springer.com/article/10.3758/s13428-017-0862-1

31. Cleeland CS, Ryan KM. Pain assessment: global use of the Brief Pain Inventory. [Internet]. Vol. 23, Annals of the Academy of Medicine, Singapore. 1994 [cited 2022 Jun 14]. p. 129–38. Available from: https://europepmc.org/article/med/8080219

32. Roelofs J, Peters ML, McCracken L, Vlaeyen JWS. The pain vigilance and awareness questionnaire (PVAQ): Further psychometric evaluation in fibromyalgia and other chronic pain syndromes. Pain. 2003 Feb 1;101(3):299–306.

33. Woby SR, Roach NK, Urmston M, Watson PJ. Psychometric properties of the TSK-11: A shortened version of the Tampa Scale for Kinesiophobia. Pain. 2005 Sep 1;117(1–2):137–44.

34. Cane D, Nielson WR, McCarthy M, Mazmanian D. Pain-related activity patterns: Measurement, interrelationships, and associations with psychosocial functioning. Clin J Pain [Internet]. 2013 May [cited 2022 Jun 14];29(5):435–42. Available from: https://journals.lww.com/clinicalpain/Fulltext/2013/05000/Pain_related_Activity

For peer review only

_Patterns__Measurement,.8.aspx

35. Sullivan MJL, Bishop SR, Pivik J. The Pain Catastrophizing Scale: Development and Validation. Psychol Assess. 1995;7(4):524–32.

36. Nicholas MK. The pain self-efficacy questionnaire: Taking pain into account. Eur J Pain. 2007 Feb 1;11(2):153–63.

37. Craig CL, Marshall AL, Sjöström M, Bauman AE, Booth ML, Ainsworth BE, et al. International physical activity questionnaire: 12-Country reliability and validity. Med Sci Sports Exerc [Internet]. 2003 [cited 2022 Jun 14];35(8):1381–95. Available from: http://www.acsm-msse.org

38. Syrjala KL, Stover AC, Yi JC, Artherholt SB, Abrams JR. Measuring social activities and social function in long-term cancer survivors who received hematopoietic stem cell transplantation. Psychooncology [Internet]. 2010 May 1 [cited 2022 Jun 27];19(5):462–71. Available from: https://onlinelibrary.wiley.com/doi/full/10.1002/pon.1572

39. Bastien CH, Vallières A, Morin CM. Validation of the insomnia severity index as an outcome measure for insomnia research. Sleep Med. 2001 Jul 1;2(4):297–307.

40. Cohen S, Kamarck T, Mermelstein R. A global measure of perceived stress. J Health Soc Behav. 1983;24(4):385–96.

41. Zigmond AS, Snaith RP. The Hospital Anxiety and Depression Scale. Acta Psychiatr Scand [Internet]. 1983 Jun 1 [cited 2018 Apr 28];67(6):361–70. Available from: http://doi.wiley.com/10.1111/j.1600-0447.1983.tb09716.x

42. Osman A, Bagge CL, Gutierrez PM, Konick LC, Kopper BA, Barrios FX. The suicidal behaviors questionnaire-revised (SBQ-R): Validation with clinical and nonclinical samples. Assessment [Internet]. 2001 Jul 26 [cited 2022 Jun

For peer review only

14];8(4):443–54. Available from: https://journals.sagepub.com/doi/abs/10.1177/107319110100800409

43. Raes F, Pommier E, Neff KD, Van Gucht D. Construction and factorial validation of a short form of the Self-Compassion Scale. Clin Psychol Psychother [Internet]. 2011 May 1 [cited 2022 Jun 14];18(3):250–5. Available from: https://onlinelibrary.wiley.com/doi/full/10.1002/cpp.702

44. Herdman M, Gudex C, Lloyd A, Janssen M, Kind P, Parkin D, et al. Development and preliminary testing of the new five-level version of EQ-5D (EQ-5D-5L). Qual Life Res [Internet]. 2011 Dec 9 [cited 2022 Jun 14];20(10):1727–36. Available from: https://link.springer.com/article/10.1007/s11136-011-9903-x

45. Carney CE, Buysse DJ, Ancoli-Israel S, Edinger JD, Krystal AD, Lichstein KL, et al. The consensus sleep diary: Standardizing prospective sleep self-monitoring [Internet]. Vol. 35, Sleep. Oxford Academic; 2012 [cited 2022 Jun 14]. p. 287–302. Available from: https://academic.oup.com/sleep/article/35/2/287/2558899

46. Ancoli-Israel S. Actigraphy. Princ Pract Sleep Med. 2005;1459–67.

47. Williams JM, Taylor DJ, Slavish DC, Gardner CE, Zimmerman MR, Patel K, et al. Validity of Actigraphy in Young Adults With Insomnia. https://doi.org/101080/1540200220181545653 [Internet]. 2018 Jan 2 [cited 2022 Jun 27];18(1):91–106. Available from: https://www.tandfonline.com/doi/abs/10.1080/15402002.2018.1545653

48. Mccall C, Mccall WV. Comparison of actigraphy with polysomnography and sleep logs in depressed insomniacs. J Sleep Res. 2012 Feb;21(1):122–7.

49. Fekedulegn D, Andrew ME, Shi M, Violanti JM, Knox S, Innes KE. Actigraphy-

For peer review only

Based Assessment of Sleep Parameters. Ann Work Expo Heal [Internet]. 2020 Apr 30 [cited 2022 Jun 27];64(4):350–67. Available from: https://academic.oup.com/annweh/article/64/4/350/5735350

50. Ancoli-Israel S, Martin JL, Blackwell T, Buenaver L, Liu L, Meltzer LJ, et al. The SBSM Guide to Actigraphy Monitoring: Clinical and Research Applications. https://doi.org/101080/1540200220151046356 [Internet]. 2015 Jul 17 [cited 2022 Jun 27];13:S4–38. Available from: https://www.tandfonline.com/doi/abs/10.1080/15402002.2015.1046356

51. Benjamini Y, Hochberg Y. Controlling the False Discovery Rate: A Practical and Powerful Approach to Multiple Testing. J R Stat Soc Ser B [Internet]. 1995 Jan 1 [cited 2022 Jun 28];57(1):289–300. Available from: https://onlinelibrary.wiley.com/doi/full/10.1111/j.2517-6161.1995.tb02031.x

52. Akaike H. Information Theory and an Extension of the Maximum Likelihood Principle. In Springer, New York, NY; 1998 [cited 2022 Jun 27]. p. 199–213. Available from: https://link.springer.com/chapter/10.1007/978-1-4612-1694-0_15

53. Akaike H. A New Look at the Statistical Model Identification. IEEE Trans Automat Contr. 1974;19(6):716–23.

54. Schwarz SG. “Estimating the Dimension of a Model.” Ann Stat [Internet]. 1978 [cited 2022 Jun 27];6(2):461–4. Available from: http://www.citeulike.org/user/abhishekseth/article/90008

55. Bates D, Mächler M, Bolker BM, Walker SC. Fitting linear mixed-effects models using lme4. J Stat Softw [Internet]. 2015 Jun 23 [cited 2022 Jun 27];67(1). Available from: https://arxiv.org/abs/1406.5823v1

56. R Core Team. R: A Language and Environment for Statistical Computing.

For peer review only

Vienna R foundation for Statistical Computing.; 2012.

57. Epskamp S, Waldorp LJ, Mõttus R, Borsboom D. The Gaussian Graphical Model in Cross-Sectional and Time-Series Data. Multivariate Behav Res [Internet]. 2018 Jul 4 [cited 2022 Jun 29];53(4):453–80. Available from: https://www.tandfonline.com/doi/abs/10.1080/00273171.2018.1454823

58. Bringmann LF, Vissers N, Wichers M, Geschwind N, Kuppens P, Peeters F, et al. A Network Approach to Psychopathology: New Insights into Clinical Longitudinal Data. PLoS One [Internet]. 2013 Apr 4 [cited 2022 Jun 27];8(4):e60188. Available from: https://journals.plos.org/plosone/article?id=10.1371/journal.pone.0060188

11. FIGURE LEGEND

Figure 1
The study uses a prospective experience sampling design involving in vivo data gathering using survey and actigraphy. The participants are asked to engage with the data collection process over 7 consecutive days (8 nights) twice, six-months apart. An example data collection procedure in a single day is detailed in the box of dashed outline. The participants are prompted to complete a sleep diary in the morning and 3 daily surveys each day. The participants are also asked to wear an actigraph during the entire 7-day (8-night) period for each wave, generating objective estimates of sleep and physical activity. The timing of the diary and surveys is pre-specified. If a participant's typical wake time is 08:00, a prompt to complete the sleep diary will be sent at 08:30, then the first, second and third daily surveys at 11:00, 13:30 and 16:00.

For peer review only

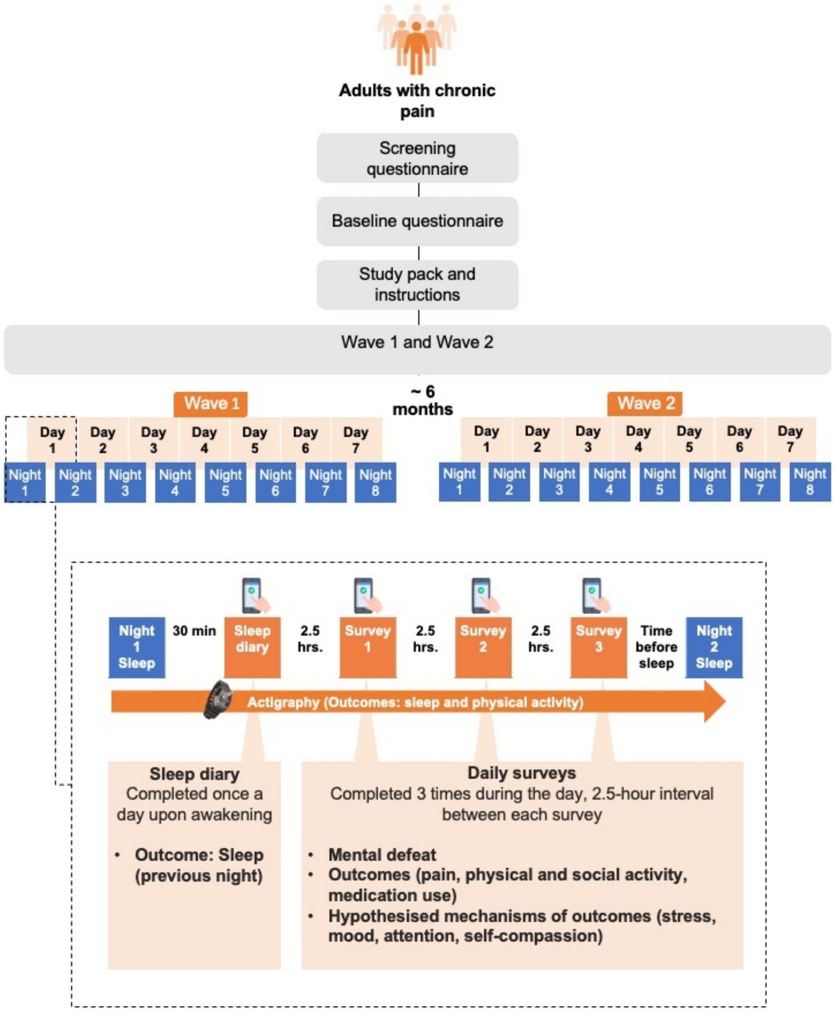

Figure 1. Study design

190x275mm (96 x 96 DPI)

COVID Checklist

The checklist is administered by a member of the research team over the telephone once per person, per timepoint (T1 and T2). This is to ensure reduced risk of virus transmission and to ensure that the sleep, pain, mood and activity measured within the study are representative of their typical chronic pain and not influenced by COVID infection. The COVID checklist requires participants to answer three questions to determine any recent exposure:

1. Have you experienced any of the main COVID-19 symptoms in the last 48 hours?

2. In the last 7 days have you come into close contact with anyone who has tested positive for COVID-19 to your knowledge?

3. Are you or is anyone in your household/bubble currently self-isolating?

If a participant answers 'yes' to one or multiple questions, they will be asked to postpone their participation until they no longer test positive. Data obtained from the checklist will not be analysed as their purposes are for health and safety and to maintain data integrity.

For peer review only

BMJ Open

Investigating Mental Defeat in Patients with Chronic Pain: Protocol for a Longitudinal Experience Sampling Study

Journal:	*BMJ Open*
Manuscript ID	bmjopen-2022-066577.R1
Article Type:	Protocol
Date Submitted by the Author:	28-Dec-2022
Complete List of Authors:	Gillett, Jenna; University of Warwick, Psychology<br>Karadag, Paige; University of Warwick, Psychology<br>Themelis, Kristy; University of Warwick, Psychology<br>Li, Yu-Mei; Bielefeld University, Psychology<br>Lemola, Sakari ; Bielefeld University, Psychology<br>Balasubramanian, Shyam; University Hospitals Coventry and Warwickshire NHS Trust<br>Singh, Swaran; University of Warwick Warwick Medical School, Mental Health and Wellbeing<br>Tang, Nicole; University of Warwick, Psychology
**Primary Subject Heading**:	Research methods
Secondary Subject Heading:	Mental health
Keywords:	PAIN MANAGEMENT, MENTAL HEALTH, STATISTICS & RESEARCH METHODS, COVID-19
Note: The following files were submitted by the author for peer review, but cannot be converted to PDF. You must view these files (e.g. movies) online.	
actiwatch_video_supplementary_material.mp4	

SCHOLARONE™ Manuscripts

TITLE: Investigating Mental Defeat in Patients with Chronic Pain: Protocol for a Longitudinal Experience Sampling Study

AUTHORS: Jenna L Gillett (PhD Researcher)[1]*, Paige Karadag (Research Assistant)[1]*, Kristy Themelis (Post-Doctoral Research Fellow)[1], Yu-Mei Li (Post-Doctoral Research Fellow)[2], Sakari Lemola (Professor)[2], Shyam Balasubramanian (Consultant)[3], Swaran Singh (Professor)[4], Nicole KY Tang (Professor)[1 a]

[1]Department of Psychology, University of Warwick, Coventry, United Kingdom

[2]Department of Psychology, Bielefeld University, Bielefeld, Germany

[3]University Hospital Coventry and Warwickshire NHS Trust, Coventry, United Kingdom

[4]Warwick Medical School, University of Warwick, Coventry, United Kingdom

*Joint first authorship (equal contributions)

[a]Corresponding Author: Nicole KY Tang, Email: n.tang@warwick.ac.uk

ORCID IDs:

J.L. Gillett: 0000-0002-7115-9938

P. Karadag: 0000-0003-2655-7109

K. Themelis: 0000-0002-0022-5272

Y.-M. Li: 0000-0002-4673-1082

S. Lemola: 0000-0002-1314-6194

S. Balasubramanian: 0000-0001-5149-3595

N.K.Y. Tang: 0000-0001-7836-9965

For peer review only

ABSTRACT

Introduction: Previous qualitative and cross-sectional research has identified a strong sense of mental defeat in people with chronic pain who also experience the greatest levels of distress and disability. This study will adopt a longitudinal experience sampling design to examine the within-person link between the sense of mental defeat and distress and disability associated with chronic pain.

Methods and Analysis: We aim to recruit 198 participants (aged 18-65 years) with chronic pain, to complete two waves of experience sampling over 1 week, 6 months apart (Time 1 and Time 2). During each wave of experience sampling, the participants are asked to complete 3 short online surveys per day, to provide in-the-moment ratings of mental defeat, pain, physical and social activity, stress, mood, self-compassion, and attention using visual analogue scales (VAS). Sleep and physical activity will be measured using a daily diary as well as with wrist-actigraphy worn continuously by participants throughout each wave. Linear mixed models and Gaussian graphical models will be fit to the data to: (1) examine the within-person, day-to-day association of mental defeat with outcomes (i.e., pain, physical/social activity, medication use and sleep) (2) examine the dynamic temporal and contemporaneous networks of mental defeat with all outcomes and the hypothesised mechanisms of outcomes (i.e., perceived stress, mood, attention, and self-compassion).

Ethics & Dissemination: The current protocol has been approved by the Health Research Authority and West Midlands – Solihull Research Ethics Committee

For peer review only

(Reference Number: 17/WM0053). The study is being conducted in adherence with the Declaration of Helsinki, Warwick Standard Operating Procedures and applicable UK legislation.

STRENGTHS AND LIMITATIONS OF THE STUDY:

- This study provides the first longitudinal investigation of mental defeat in chronic pain to shed light on its temporal links with outcomes.
- A range of outcomes and hypothesised mechanisms will be assessed including pain, physical and social activity, medication use, sleep, stress, mood, attention, and self-compassion. Measures are repeated over a one-week period, at two time-points each six-months apart.
- This study will use both self-reported and objective estimates of sleep and physical activity, via diaries and actigraphy longitudinally.
- The research is done remotely, at the participant's convenience and within their natural environment.
- Considerations must be given to effects of participants' COVID exposure on recruitment, subsequent attrition, and possible findings despite having had appropriate COVID screening and health and safety procedures in place.

For peer review only

1. INTRODUCTION

Chronic pain is characterised as pain that persists or recurs beyond 3 months (1). It is highly prevalent, affecting around 30% of the population worldwide (2–4). Chronic pain conditions, namely low back pain and headaches, are consistently the top causes of years lived with disability (2,5) and reduced quality of life (6). People with chronic pain are three times as likely to have depression and anxiety disorders (7) and twice as likely to present a risk of suicide compared to the general population (8). Whilst some individuals manage to cope with the pain, others struggle to maintain daily activities. Understanding the factors that determine whether an individual can feel and function well- despite persistent pain- is crucial to advancing non-pharmacological management approaches for chronic pain, which have so far had a modest impact (9).

A concept proposed to help explain differences in the experience of pain-related distress and disability is mental defeat; a cognitive construct characterised by negative self-appraisals in relation to pain (10,11). The concept of defeat has its theoretical underpinnings in the study of post-traumatic stress disorder (PTSD) (12–14) and depression (15,16), where it is respectively defined as the perceived loss of autonomy and a natural response to the loss of social status in a conflict situation. Empirical research has shown that a strong sense of mental defeat is associated with severe PTSD symptoms and poorer response to exposure treatment (12,14,17). The perception of defeat has also been shown to predict symptoms of depression independent of hopelessness (15), and has been implicated in psychological models of suicidal behaviour and suicidality (18–20).

Mental defeat in the context of chronic pain encapsulates people's psychological response to perceived threats of one's physical and psychological

For peer review only

autonomy. Daily experience of living with persistent and debilitating pain which does not respond to treatment is thought to be a repeated trigger of mental defeat, prompting negative appraisals of self in relation to pain (21). Consistently, qualitative explorations of patient experience have revealed patients reporting “defeat of the mind” and “the pain is taking over”, with the pain seen as “an enemy” that “belittles [them] as a person” (11,22,23).

Using the Pain Self Perception Scale to measure mental defeat, it has been found that treatment-seeking patients with chronic pain have elevated levels of mental defeat compared to: patients with acute pain, patients with anxiety disorders, community volunteers with chronic pain, community volunteers with acute pain and pain-free volunteers (21). Mental defeat has also been found to be the predictor explaining the most variance in pain interference, depression, and psychological disability among chronic pain patients seeking specialist treatment, when compared to pain intensity, health anxiety, worry rumination, and pain catastrophising (11). It has moderate associations with sleep disturbances and functional disability (11) and negatively relates to pain self-efficacy even when anxiety, depression, pain catastrophising, and hopelessness are controlled for (24). Furthermore, mental defeat predicts suicide intent in patients with chronic pain above and beyond pain intensity (25). In pain-free volunteers, an activated sense of mental defeat appears to operate independently from existing pain-related psychological constructs such as pain catastrophising, in influencing mood and attentional disengagement from nociceptive stimuli (26). Together, these findings suggest how a person’s self-perception in relation to pain matters in terms of predicting and explaining outcomes.

However, most of the aforementioned studies of mental defeat in chronic pain are cross-sectional in design, and more direct evidence is required to establish the

For peer review only

temporal and casual association implicated. This study will be the first to utilise experience sampling methodology (ESM) involving *in vivo* data gathering (using actigraphy, sleep diaries and daily online surveys) to examine the day-to-day association between mental defeat, symptoms, distress, and disability associated with chronic pain. Data will be collected over 7 days, allowing the study of any temporal within-person relationships which may provide insight into clinically relevant questions such as whether mental defeat will be followed by higher pain, increased stress, greater attention to pain, increased medication usage, reduced physical and social activity and poorer sleep. The experience sampling exercise is repeated at 6 months to investigate how these indices may change over time and translate into distress and disability long-term. The primary objectives of this study are: (1) examine the within-person, day-to-day association of mental defeat with outcomes (i.e., pain, physical/social activity, medication use and sleep), and (2) examine the dynamic temporal and contemporaneous networks of mental defeat with all outcomes and the hypothesised mechanisms of outcomes (i.e., perceived stress, mood, attention, and self-compassion).

Based on previous work (e.g. 11,21,25) we hypothesise that, for an individual, a strong sense of mental defeat will be associated with subsequent greater reports of pain, reduced physical and social activity, possible increased use of medication, and poorer sleep. Examinations of the dynamic temporal and contemporaneous networks of mental defeat with mechanisms and outcomes are novel and exploratory.

2. METHOD

2.1 Study Design

For peer review only - http://bmjopen.bmj.com/site/about/guidelines.xhtml

This study utilises a within-study design that uses an experience sampling methods (ESM) approach (27). As depicted in Figure 1, ESM are used to gather data prospectively over 1-week; at two-time points, each six months apart (T1 and T2). The length and frequency of assessment reflect our attempt to balance information needs with concerns of participation burden and possible attrition, participants are asked to continuously wear a medical-grade actigraphy device (MotionWatch 8©, manufactured by CamNTech Ltd) for 8 nights/7 days. They are asked to complete a sleep diary and respond to 3 short online surveys during the day. These surveys will provide in-the-moment measures of mental defeat, pain, medication use, physical activity and social activity, stress, mood, attention, and self-compassion using visual analogue scales (VAS).

Insert Figure 1 about here

2.2 Participants & Sample Size

Participants are adults aged 18-65 living with chronic non-cancer pain that has been present or recurring for more than 3 months (28,29). Participant inclusion and exclusion criteria are outlined in Table 1.

Table 1.
PARTICIPANT INCLUSION & EXCLUSION CRITERIA FOR PARTICIPATION

Inclusion Criteria:
➢ Aged between 18 – 65 (for focussing our study on working-age population).<br>➢ Experience chronic non-cancer pain for at least 3 months.<br>➢ Stable treatment for duration of the study (6 months).<br>➢ English-speaking (for understanding and implementing the data collection procedure).<br>➢ Living in the UK (for postage of equipment).<br>➢ Be able to provide informed consent.

For peer review only

Exclusion Criteria:
➢ Have any significant comorbid psychiatric (e.g., psychosis), medical (e.g., coronary heart diseases), neurological (e.g., Alzheimer's, Parkinson's, Epilepsy) or life-threatening conditions that would impact pain experience, impede the ability to provide informed consent or complete the study.<br>➢ Have any other significant comorbid sleep disorder, e.g., sleep apnoea, restless leg syndrome, periodic limb movement disorder, narcolepsy or circadian rhythm disorders, which in the opinion of the research team would cofound the results of the study.<br>➢ Have elective surgery or procedures requiring general anaesthetic during the study.<br>➢ Have participated in another research study using an investigational product in the past 3-months.

**Note: the examples given in the inclusion/exclusion criteria are not exhaustive and participants' eligibility is assessed on a case-by-case basis by the research team.*

In terms of sample size calculation, based on running previous ESM studies of this kind (30) we aim to recruit 198 participants to factor in an anticipated 20% attrition at T2. This will give an estimated 158 participants who will complete the experience sampling procedure at both timepoints to generate up to 6636 temporally structured survey ratings for analysis (3 ratings x 7 days x 2 timepoints x 158 participants). There will be a maximum of 2528 observations of sleep data (8 nights of actigraphic data x 2 timepoints x 158 participants) and 7 days' of physical activity count data at 30-second epoch. This will give sufficient power to perform the planned analyses using Multilevel Mixed-Effects Models (31) and Graphical Gaussian Models (32).

2.3 Recruitment

Recruitment of participants started in April 2021 and is expected to end in May 2023. A variety of recruitment methods are being used, including social media, the NIHR Clinical Research Network, public engagement events, and peer-led support groups. We are also using online recruitment platforms to capture individuals with chronic pain with registered interest to take part in research. Finally, chronic pain patients at

For peer review only

University Hospitals Coventry and Warwickshire are given information about the study during pain clinic appointments.

2.4 Measures

2.4.1 Screening Questionnaire

A brief online screening questionnaire is administered to assess eligibility. To provide the relevant information against the *a priori* inclusion and exclusion criteria, the screening questionnaire determines basic demographics (e.g., age, sex, ethnicity, employment status, education level) and health indicators (e.g., body mass index, average alcohol intake, smoking status). It also checks for pain characteristics, current treatment plans, e.g. plans for surgery in the next 6-months and current participation in clinical trials. Lastly, it considers comorbid health conditions, including the presence of any psychiatric, medical, neurological or sleep disorders.

2.4.2 Baseline Questionnaire

Eligible participants are asked to complete a baseline questionnaire which includes validated measures of variables related to mental defeat, pain, physical and social activity, sleep, psychological states and quality of life (see Table 2 for full list). Data from these questionnaires are not used in the planned analyses of the current study except for characterising the sample at baseline.

Table 2.
QUESTIONNAIRE MEASURES INCLUDED

Measure	Scale Used
**Key Variable of Interest**	
Mental defeat	*Pain Self-Perception Scale (PSPS)*(21)
**Pain-Related Measures**	

For peer review only

Pain intensity & interference	*Brief Pain Inventory-Short Form (BPI-sf)*(33)
Pain vigilance and awareness	*Pain Vigilance & Awareness Questionnaire (PVAQ)*(34)
Pain-related fear of movement	*Tampa Scale of Kinesiophobia (TSK-11)*(35)
Patterns of activity (pain-specific)	*Patterns of Activity Measure for Pain (POAM-P)*(36)
Pain catastrophising	*Pain Catastrophizing Scale (PCS)*(37)
Pain self-efficacy	*Pain Self-Efficacy Questionnaire (PSEQ)*(38)
**Physical & Social Activity Measures**	
Physical activity	*International Physical Activity Questionnaire (IPAQ)*(39)
Social activity	*Social Activity Log (SAL)*(40)
**Sleep-Related Measure**	
Insomnia symptom severity	*Insomnia Severity Index (ISI)*(41)
**Psychological States**	
Stress	*Perceived Stress Scale (PSS)*(42)
Anxiety & depression	*Hospital Anxiety & Depression Scale (HADS)*(43)
Suicidal behaviour	*Suicidal Behaviour Questionnaire Revised (SBQ-R)*(44)
Self-compassion	*Self-Compassion Scale Short Form (SCS-sf)*(45)
**Quality of Life Measure**	
General health and quality of life	*EQ-5D-5L*(46)

2.4.3 Sleep Diary

The morning section of the Consensus Sleep Diary (47) is administered daily throughout the tracking period to collect self-reported information on sleep. The sleep diary asks participants what time they went to bed, what time they attempted to go to sleep, how long it took them to fall asleep (sleep onset latency), how many times did they wake up from sleep (not including final awakening), final wake up time, what time did they get out of bed, total sleep duration (hours and minutes), perceived sleep-quality and how rested or refreshed they felt after waking. Participants can also provide additional information that they feel is relevant to their

For peer review only

sleep. We added two extra questions to the sleep diary to obtain in-the-moment ratings of pain and mood upon waking. These possible covariates are assessed via two VAS both ranging from 0-10, whereby for pain 0= *no pain at all* and 10= *worst pain imaginable* and for mood 0= *very bad* and 10= *very good*.

2.4.4 Daily Survey

The daily survey allows participants to provide self-report ratings at multiple points throughout each day. We use adapted or proxy measures as well as shortened versions of original scales to decrease participant burden and determine momentary assessments of mental defeat, pain, medication use, physical and social activity, stress, mood, attention, and self-compassion (see Figure 1 for a schedule of administration). As part of the PPI piloting process, the selected questions and proxy measures were approved by our PPI representatives (two people with lived experience of chronic pain). The surveys are short (<5 minutes completion time) and are equally spaced 2.5 hours apart to capture experiences at different time points throughout the day. The surveys are sent out following a choice of pre-determined schedules to match participants' typical sleep-wake patterns. The earliest schedule starts with a sleep diary at 06:30 and the first daily survey commences at 09:00, whereas the latest schedule starts with a sleep diary at 13:30 and the first daily survey commences at 16:00. The timing of these measures avoids unsociable hours, as no prompts arrive between the hours of 23:00 and 06:00 inclusive.

We use Survey Signal to send out auto-prompts at specified times via SMS to participants' smartphones. This enables accessibility for participants to complete surveys in a timely fashion, while remaining convenient, and does not require an app download or adjusted personal mobile settings. The surveys are administered,

For peer review only

recorded, and returned via Qualtrics and are time-stamped at the time of commencement and completion. The daily surveys comprise multiple VAS with varying left to right anchors, as shown in Table 3.

Table 3.
DAILY SURVEY SELF-REPORT RATING SCALES

Construct	Item (measure)	Scale	Anchors
Mental defeat	Since waking up today, how much has the pain brought back to life memories of times when you felt the pain had taken over?	0 – 10	0= not at all to 10= very much so
Pain intensity	What is your current pain level?	0 – 10	0 = no pain to 10 = worst pain imaginable
Pain interference	Since waking up today, how much has your pain impacted on…<br>a) Your daily routine (including work)?<br>b) Your relationship(s)?<br>c) How you think or feel about the future?<br>d) How you think or feel about yourself?	0 – 10	0= not much impact/interference to 10 = a great deal of impact/interference
Medication use	Since waking up today, would you say that you have taken more or less medication than usual?	-5 – 5	-5= a lot less than usual, 0 = no difference, 5 = a lot more than usual
Physical activity	Since waking up today, how physically active have you been?	0 – 10	0= not physically active at all to 10= very physically active
Social activity	Since waking up today, how socially engaged have you been?	0 – 10	0= not socially engaged at all to 10= very socially engaged
Stress	What is your current stress level?	0 – 10	0= no stress at all to 10= highest level of stress possible
Mood	What is your current mood?	0 – 10	0= very bad to 10 = very good

For peer review only

Attention to pain	Since waking up today, how much of the time have you been thinking about your pain?	0 – 10	0= none of the time to 10= a great deal of the time
Focus of attention	Since waking up today, has the focus of your attention been… a) Inward or outward b) On the body or mind	0 – 10	0 = inward to 10 outward 0 = body to 10= mind
Self-compassion	Since waking up today, how kind… a) to yourself have you been? b) to others have you been?	0 – 10	0= not at all to 10= very much so

Note. VAS questions with corresponding anchors that are presented in Survey 1 are summarised above. These questions appear identically in Surveys 2 and 3, except instead of starting questions with "since waking up today…" in Surveys 2 and 3 each question begins "in the last 2.5 hours…".

2.4.5 Actigraphy

Actigraphs are light, compact accelerometer-based devices that have been used to generate objective estimates of sleep parameters for several decades (48). Actigraphy has been well-evidenced as a suitable methodology for non-intrusive at-home sleep assessment (49–51). Although polysomnography continues to be considered the gold standard for sleep recording, wrist-actigraphy has the advantage of offering cost-effective continuous recording in participants' home environment (52). Thus providing more ecologically valid information compared to polysomnography (52).

For the present study, MotionWatch 8© actigraphs are worn by participants on their non-dominant wrist during the study. The MotionWatch is a medical-grade tri-axial actigraphy device containing a piezoelectric accelerometer to record duration, integration, and number of movements in all directions. This data enables the research team to chart sleep and physical activity across the experience sampling

For peer review only

periods that are then downloaded for analysis using MotionWare© software (Cambridge Neurotechnology Ltd., Cambridge, UK) with validated algorithms. The key sleep parameters we are interested in are sleep efficiency (SE) and total sleep time (TST). The key physical activity parameter is total activity counts tabulated by week, day, and/or hour.

2.5 Procedure

To participate, individuals respond to a study advert via phone, email or by following a direct link to the information leaflet. Interested participants are required to complete the screening questionnaire and contact information form, following which a member of the research team determines eligibility to the study by checking against the inclusion/exclusion criteria. Individuals who meet the inclusion criteria are informed of their eligibility via email and invited to participate. Individuals who do not meet the inclusion criteria are informed via email that they are not eligible, thanked and debriefed.

Eligible participants that agree to participate are sent a link to an online consent form to complete via Qualtrics. Once informed consent has been obtained, participants have a phone call with a member of the research team to arrange their participation and highlight some key training points for the study. All participants receive the study materials (an invitation letter, a cleaned and packaged MotionWatch for borrowed use, two individually wrapped disinfectant wipes, an addressed, pre-paid return envelope, and an information booklet) via UK postal delivery. To accompany the information booklet, participants are emailed a link to an instructional video (see supplementary file 1) demonstrating how to use the MotionWatch devices appropriately and reiterating the schedule of

For peer review only

measurement/engagement required during participation. Participants are instructed to wear the MotionWatch continuously for the 7-day experience sampling period and are required to press an event marker on the device when they plan to go to sleep and when they get out of bed, following their main sleep period. Participants are also asked to answer the sleep diary and three short surveys each day.

The timing of the daily surveys/sleep diary are individually anchored by participants' typical sleep-wake patterns, to accommodate and control for variations in individuals' circadian rhythms. For example, if a participant indicates that they usually wake at 08:00, a typical day during the experience sampling period on this schedule would be as follows: upon getting out of bed, the participant presses the event marker on the MotionWatch and will receive the first text message (with a link to the sleep diary) at 08:30 – allowing them to report their sleep experiences as soon as practical after waking. Throughout the day, the participant will receive three further text message prompts, each containing a link to the short online survey. Survey 1 (S1) is received at 11:00, survey 2 (S2) at 13:30 and survey 3 (S3) at 15:00. The links to each survey remains open for 2.5 hours, before expiring at the time the following survey is triggered. Finally, the participant will be required to press the event marker at "lights out" or when beginning trying to sleep. This process is identical for each day in the experience sampling period. After the final awakening on the last day, the participant removes the MotionWatch, and packages it in the box ready for return-postage to the Lab. The MotionWatch data is processed on-site at the Lab, before being formatted into a personalised breakdown for the participant, which is emailed to them along with a gift voucher within two weeks of receipt of the returned equipment. This procedure is repeated in its entirety for the follow-up T2 assessment. The debrief is administered upon study completion.

For peer review only

2.6 Participant Reimbursement

To thank the participants for their time and participation, they are given a £10 gift voucher for each timepoint they complete. Additionally, participants are provided with a personalised breakdown of their actigraphy data created by the research team. No evaluative feedback on sleep quality and physical activity patterns is given.

2.7 Adverse Event Recording and Management

This is a low-risk observational study, and no major adverse events are anticipated. We offer health and safety training at the outset, instructing participants not to respond to survey text messages if it is not safe to do so, e.g., when driving, operating machinery, or crossing the road. Before participation commences, participants are instructed to report any adverse events that occur during the assessment periods to the research team.

Adverse events that are related to the study and/or unrelated adverse events are recorded and reported to the study sponsor according to reporting requirements. Unrelated and expected adverse events may include but are not limited to illness, hospitalisation or day-surgery that occurs during the assessment period. An adverse event that a small number of participants may experience is discomfort or irritation caused by wearing the MotionWatch. Before participants begin the study, we advise them to inform the researcher if they experience any skin irritation. If the irritation is very mild and they wish to continue, we recommend placing a small piece of tissue underneath the watch or to place the silicon strap on top of their sleeve to avoid direct skin contact. In the unlikely event of an unexpected adverse event that is deemed to be severe and related to the study, the research team would immediately

For peer review only

pause the study and send an expedited report to the study sponsor. Events will be followed up until they are resolved or when a final outcome has been reached.

2.8 Patient and Public Involvement

This protocol has been developed in partnership with two patient representatives with lived experience of chronic pain. One representative (PR) provided feedback on participant-facing materials and piloted the MotionWatch for at-home use. DD participated in study-related procedures and also commented on the manuscript for readability.

2.9 COVID Related Considerations

To enable the study to take place during the COVID-19 pandemic, while adhering to governmental and institutional COVID-19 guidance, the operational aspects of the protocol have been adapted for remote participation. The MotionWatch devices are all sanitised, prepared and packaged for participation in a lab environment. A short COVID checklist has been implemented before each wave of tracking (see supplementary file 2).

3. DATA MANAGEMENT PLAN

The study data collected from the questionnaire, sleep diary and daily surveys will be stored securely on Qualtrics and subsequently downloaded as password protected databases to undergo data quality checks and pseudo-anonymisation by the research team. Access to these databases will be restricted to approved members of the research team. Once data completeness and quality are verified, electronic data on Qualtrics will be deleted.

For peer review only

Actigraphic data collected using MotionWatch 8© are downloaded using Motionware© software upon each watch's return to the lab. The downloaded data are saved via their assigned ID number and will be held securely and separately from the study data.

The Chief Investigator (NT) of the project will act as the data controller. All data generated by the research programme will be analysed by the research team either on site at the University of Warwick or in a private workspace in the event of remote-working, as per the University's Off-Campus Working Policy. In line with the University's Research Code of Conduct, data will be retained in electronic format for at least 10 years from the date of any publication that is based upon it.

4. STATISTICAL METHODS

All participants who meet eligibility criteria will be included in analyses; including those who wish to withdraw from the study but consent to having any data already collected analysed. Those who withdraw and do not consent to the data being analysed will be excluded from analyses.

Descriptive statistics will be used to characterise the sample based on information from the screening and baseline questionnaires. Means and standard deviations/95% confidence intervals will be reported for continuous variables, whereas frequencies and percentages will be used for reporting categorical variables.

To evaluate the within-person temporal relationship between mental defeat (predictor) and pain, physical and social activity, medication use and sleep (outcomes), we will pool the daily survey ratings from all participants across waves of assessment. Linear mixed models with a time-lagged design will be fit to the data.

For peer review only

We will fit one model for each outcome. For each analysis, we will first estimate a maximal random effects model with all random slopes and random intercepts (for ‘time of day [survey 1, 2, 3]’, ‘day [1, 2, 3, 4, 5, 6, 7]’ and ‘wave [1, 2]’) and successively simplify the random effect structure until the model converges. Nested comparisons will be made between the final models with the intercepts-only models. The significance of each model will be assessed using a likelihood ratio test (LRT). P-values will be adjusted using false discovery rate to account for alpha error accumulation (FDR; (53)). The best fitting model will be determined using Aikaike Information Criteria (AIC) (54,55) and Bayesian Information Criteria (BIC) (56), where lower values of the AIC and BIC indicate a better fit. As secondary analyses, we will repeat the above analyses with the hypothesised mechanisms of outcomes (stress, mood, attention, self-compassion) as the dependent variables. Both the statistical software SPSS (IBM Corp., Armonk, NY, USA) and the “lme4” package (57) for R (58) will be used.

To explore the within-individual temporal and contemporaneous relationships as well as between-individual relationships between mental defeat and the outcomes and hypothesised mechanisms of interest, we will use Gaussian graphical models (GGM; (32)). To obtain sufficient statistical power for within-in individual variances in the GGMs, it is recommended to have more than 20 measurements and at least 20 pairs of comparisons per participant (59). The design of the current study will yield 21 measurements per participant per wave. That will give 20 pairs of comparisons for modelling changes across days, 14 pairs of comparisons for modelling changes within day, and 7 pairs of comparisons for modelling changes at different times of the day. We will therefore not seek to model changes at different times of the day, but to

For peer review only

focus on modelling changes across and within day by pooling together data from both waves of assessment.

We will use the mlVAR (Version 0.3.2) package (59,60), or similarly suitable but more up-to-date packages, for R (58) to analyse the multivariate time series data. We will report three kinds of network; temporal (to indicate whether a variable predicts another variable (or itself) at the next measurement point, controlling for all other variables in the network at the previous measurement point), contemporaneous (to indicate the within-person relationships between variables, having adjusted for the effect of all variables in the network and the temporal effects) and between-person (to indicate relationships between person-means of variables, partialling out the effect of all other variables in the network).

Additional secondary analyses will be performed for research questions other than the 2 main ones stated here. Any deviation(s) from the original statistical plan will be described and justified in our subsequent reportings.

5. LIMITATIONS

Although this study design provides the first prospective investigation of mental defeat in chronic pain, more research is needed to evidence any potential causality of key mechanisms and outcomes. As in previous experience sampling studies that measure sleep, we expect some minor discrepancies between actigraphy and diary data (61) due to memory biases when self-reporting on one's own sleep estimations, which is common in this type of research. In this study we will cross-check for any discrepancies between diary and objective sleep measures and will run sensitivity analyses to determine effective interpretation in subsequent analyses. Furthermore, participants are required to undergo some short training on how to participate in the

For peer review only

study and use the equipment (actigraphy devices) effectively, which will naturally result in some errors and inconsistencies in individual engagement levels, so we are expecting some data to be lost to missingness. Finally, considerations must be given to effects of participants' COVID exposure on recruitment, subsequent attrition, and possible findings despite having had appropriate COVID screening and health and safety procedures in place.

6. ETHICS & DISSEMINATION

This research forms part of the wider MRC-funded Warwick Study of Mental Defeat in Chronic Pain ("WITHIN" Study). The current protocol has been approved by the Health Research Authority and West Midlands – Solihull Research Ethics Committee (Reference Number: 17/WM0053, p, IRAS project ID: 223190). The University of Warwick (Research Impact Services, University of Warwick, Coventry, CV4 7AL) acts as the Sponsor for the study. The study is being conducted in adherence with the Declaration of Helsinki, Warwick Standard Operating Procedures and applicable UK legislation. Results from this study will be written as reports, to be disseminated in peer-reviewed journals, at conferences and patient and public engagement events.

7. AUTHOR'S CONTRIBUTIONS AND ACKNOWLEDGEMENTS

6.1 Authors' Contributions

NT conceived the research idea and developed the theory and plan for this study. JG and PK are joint first author as they contributed equally to all aspects of the study and are responsible for implementing the protocol, creating study materials, data acquisition and management and drafting the original manuscript. KT and NT are

For peer review only

responsible for critical revisions of the manuscript. All authors (JG, PK, KT, Y-ML, SL, SB, SS, NT) contributed to the study development and reviewed, commented, and approved the manuscript.

6.2 Acknowledgements

The authors would like to thank all the participants who are involved in the study and the UK chronic pain charities who have shared our study on relevant webpages and social media platforms. We would also like to thank our PPI representatives (PR & DD) for their input into the study design and feasibility, including Debra Dulake (DD) who also commented on the manuscript. Finally, we would like to thank Michelle Pun, Emily Ashton and Chloe Osei-Cobbana who have supported the data collection process under the Psychology Research Skills Development Scheme (PRSDS) at the University of Warwick.

8. COMPETING INTERESTS

The authors have no competing interests.

9. FUNDING

This protocol is part of a wider study (Warwick Study of Mental Defeat in Chronic Pain that is supported by Medical Research Council (MRC) grant number: MR/S026185/1 to NKYT. For the purpose of open access, the corresponding author has applied a Creative Commons Attribution (CC BY) licence (where permitted by UKRI, ‘Open Government Licence’ or ‘Creative Commons Attribution No-derivatives (CC BY-ND) licence’ may be stated instead) to any Author Accepted Manuscript version arising from this submission. JLG was funded by the University of Warwick

For peer review only

Departmental PhD Fellowship, under the supervision of NKYT. The funders have no role in study design, data collection and analysis, decision to publish or preparation of this manuscript.

10. DATA SHARING STATEMENT

Once available, cleaned, anonymised data generated from this study will be made available on open-access repository.

11. REFERENCES

1. Treede RD, Rief W, Barke A, Aziz Q, Bennett MI, Benoliel R, et al. Chronic pain as a symptom or a disease: The IASP Classification of Chronic Pain for the International Classification of Diseases (ICD-11) [Internet]. Vol. 160, Pain. Lippincott Williams and Wilkins; 2019 [cited 2022 Jun 14]. p. 19–27. Available from: https://journals.lww.com/pain/Fulltext/2019/01000/Chronic_pain_as_a_symptom_or_a_disease__the_IASP.3.aspx
2. James SL, Abate D, Abate KH, Abay SM, Abbafati C, Abbasi N, et al. Global, regional, and national incidence, prevalence, and years lived with disability for 354 diseases and injuries for 195 countries and territories, 1990–2017: a systematic analysis for the Global Burden of Disease Study 2017. Lancet. 2018 Nov 10;392(10159):1789–858.
3. Dahlhamer J, Lucas J, Zelaya, C, Nahin R, Mackey S, DeBar L, et al. Prevalence of Chronic Pain and High-Impact Chronic Pain Among Adults — United States, 2016. Morb Mortal Wkly Rep [Internet]. 2018 Sep 9 [cited 2022 Jun 14];67(36):1001–6. Available from: /pmc/articles/PMC6146950/

For peer review only

4. Cohen SP, Vase L, Hooten WM. Chronic pain: an update on burden, best practices, and new advances [Internet]. Vol. 397, The Lancet. Elsevier; 2021 [cited 2022 Jun 14]. p. 2082–97. Available from: http://www.thelancet.com/article/S0140673621003937/fulltext
5. Vos T, Abajobir AA, Abbafati C, Abbas KM, Abate KH, Abd-Allah F, et al. Global, regional, and national incidence, prevalence, and years lived with disability for 328 diseases and injuries for 195 countries, 1990-2016: A systematic analysis for the Global Burden of Disease Study 2016. Lancet [Internet]. 2017 Sep 16 [cited 2022 Jun 29];390(10100):1211–59. Available from: https://research.polyu.edu.hk/en/publications/global-regional-and-national-incidence-prevalence-and-years-lived
6. Fernández A, Saameño JÁB, Pinto-Meza A, Luciano JV, Autonell J, Palao D, et al. Burden of chronic physical conditions and mental disorders in primary care. Br J Psychiatry [Internet]. 2010 Apr [cited 2022 Jun 14];196(4):302–9. Available from: https://www.cambridge.org/core/journals/the-british-journal-of-psychiatry/article/burden-of-chronic-physical-conditions-and-mental-disorders-in-primary-care/1F431966FA03440CA86EE7B2B2028FB6
7. Von Korff M, Crane P, Lane M, Miglioretti DL, Simon G, Saunders K, et al. Chronic spinal pain and physical-mental comorbidity in the United States: Results from the national comorbidity survey replication. Pain [Internet]. 2005 Feb [cited 2022 Jun 28];113(3):331–9. Available from: https://journals.lww.com/pain/Fulltext/2005/02000/Chronic_spinal_pain_and_physical_mental.13.aspx
8. Tang NKY, Crane C. Suicidality in chronic pain: A review of the prevalence, risk factors and psychological links [Internet]. Vol. 36, Psychological Medicine.

For peer review only

Cambridge University Press; 2006 [cited 2022 Jun 14]. p. 575–86. Available from: https://www.cambridge.org/core/journals/psychological-medicine/article/abs/suicidality-in-chronic-pain-a-review-of-the-prevalence-risk-factors-and-psychological-links/D3093AA52A611348B8F90CF0972380C5

9. Williams AC d. C, Fisher E, Hearn L, Eccleston C. Psychological therapies for the management of chronic pain (excluding headache) in adults [Internet]. Vol. 2020, Cochrane Database of Systematic Reviews. John Wiley and Sons Ltd; 2020 [cited 2022 Jun 14]. Available from: https://www.cochranelibrary.com/cdsr/doi/10.1002/14651858.CD007407.pub4/full

10. Tang NKY, Salkovskis PM, Hanna M. Mental Defeat in Chronic Pain: Initial Exploration of the Concept. Clin J Pain [Internet]. 2007 Mar [cited 2020 Apr 16];23(3):222–32. Available from: http://journals.lww.com/00002508-200703000-00005

11. Tang NKY, Goodchild CE, Hester J, Salkovskis PM. Mental defeat is linked to interference, distress and disability in chronic pain. Pain. 2010 Jun 1;149(3):547–54.

12. Ehlers A, Maercker A, Boos A. Posttraumatic stress disorder following political imprisonment: The role of mental defeat, alienation, and perceived permanent change. J Abnorm Psychol [Internet]. 2000 [cited 2022 Jun 28];109(1):45–55. Available from: /record/2000-13847-006

13. Dunmore E, Clark DM, Ehlers A. A prospective investigation of the role of cognitive factors in persistent Posttraumatic Stress Disorder ({PTSD}) after physical or sexual assault. Behav Res Ther. 2001 Sep;39(9):1063–84.

14. Dunmore E, Clark DM, Ehlers A. Cognitive factors involved in the onset and

For peer review only

maintenance of posttraumatic stress disorder (PTSD) after physical or sexual assault. Behav Res Ther. 1999 Sep 1;37(9):809–29.

15. Gilbert P, Allan S. The role of defeat and entrapment (arrested flight) in depression: An exploration of an evolutionary view. Psychol Med [Internet]. 1998 [cited 2022 Jun 14];28(3):585–98. Available from: https://www.cambridge.org/core/journals/psychological-medicine/article/abs/role-of-defeat-and-entrapment-arrested-flight-in-depression-an-exploration-of-an-evolutionary-view/A28C75514B33418F797269B1C276AC87

16. Price J, Sloman L, Gardner R, Gilbert P, Rohde P. The social competition hypothesis of depression [Internet]. Vol. 164, British Journal of Psychiatry. Cambridge University Press; 1994 [cited 2022 Jun 14]. p. 309–15. Available from: https://www.cambridge.org/core/journals/the-british-journal-of-psychiatry/article/abs/social-competition-hypothesis-of-depression/18622D076771AB200A26D318AF52A1D9

17. Ehlers A, Clark DM, Dunmore E, Jaycox L, Meadows E, Foa EB. Predicting response to exposure treatment in PTSD: The role of mental defeat and alienation. J Trauma Stress [Internet]. 1998 Jul 1 [cited 2022 Jun 28];11(3):457–71. Available from: https://onlinelibrary.wiley.com/doi/full/10.1023/A%3A1024448511504

18. Williams M. Cry of Pain: Understanding Suicide and the Suicidal Mind [Internet]. 2014 [cited 2022 Jun 14]. 304 p. Available from: https://books.google.co.uk/books?hl=en&lr=&id=msamAgAAQBAJ&oi=fnd&pg=PT6&dq=+Williams,+M.+(2014).+Cry+of+pain:+understanding+suicide+and+the+suicidal+mind.+Hachette+UK.&ots=ssXWHmmFa7&sig=k3HlS7rgmKO0C

For peer review only

XiZiu5yLb4FkBA&redir_esc=y#v=onepage&q=Williams%2C M.

19. O'Connor RC, Kirtley OJ. The integrated motivational-volitional model of suicidal behaviour [Internet]. Vol. 373, Philosophical Transactions of the Royal Society B: Biological Sciences. The Royal Society; 2018 [cited 2022 Jun 14]. Available from: https://royalsocietypublishing.org/doi/10.1098/rstb.2017.0268

20. Johnson J, Gooding PA, Wood AM, Tarrier N. Resilience as positive coping appraisals: Testing the schematic appraisals model of suicide (SAMS). Behav Res Ther [Internet]. 2010 Mar 1 [cited 2020 Mar 25];48(3):179–86. Available from: https://reader.elsevier.com/reader/sd/pii/S0005796709002484?token=047BFC4DC2AA4BAC774ED9741F8C3365F5796A2CDB6555ABD4E520173D205170BFF3942B9E193578519667E13A37C052&originRegion=eu-west-1&originCreation=20211006105621

21. Tang NKY, Salkovskis PM, Hanna M. Mental defeat in chronic pain: Initial exploration of the concept. Clin J Pain [Internet]. 2007 Mar [cited 2022 Jun 14];23(3):222–32. Available from: https://journals.lww.com/clinicalpain/Fulltext/2007/03000/Mental_Defeat_in_Chronic_Pain__Initial_Exploration.5.aspx

22. Smith JA, Osborn M. Pain as an assault on the self: An interpretative phenomenological analysis of the psychological impact of chronic benign low back pain. Psychol Heal [Internet]. 2007 Jul [cited 2022 Jun 14];22(5):517–34. Available from: https://www.tandfonline.com/doi/abs/10.1080/14768320600941756

23. Tang NKY, Salkovskis PM, Hodges A, Soong E, Hanna MH, Hester J. Chronic pain syndrome associated with health anxiety: A qualitative thematic

For peer review only

comparison between pain patients with high and low health anxiety. Br J Clin Psychol [Internet]. 2009 Mar 1 [cited 2022 Jun 14];48(1):1–20. Available from: https://onlinelibrary.wiley.com/doi/full/10.1348/014466508X336167

24. Hazeldine-Baker CE, Salkovskis PM, Osborn M, Gauntlett-Gilbert J. Understanding the link between feelings of mental defeat, self-efficacy and the experience of chronic pain. Br J Pain [Internet]. 2018 May 16 [cited 2022 Jun 14];12(2):87–94. Available from: http://www.ncbi.nlm.nih.gov/pubmed/29796260

25. Tang NKY, Beckwith P, Ashworth P. Mental defeat is associated with suicide intent in patients with chronic pain. Clin J Pain. 2016;32(5):411–9.

26. Collard VEJ, Gillett JL, Themelis K, Tang NKY. An exploratory investigation into the effects of mental defeat on pain threshold, pain rating, pain anticipation, and mood. Curr Psychol [Internet]. 2021 Mar 10 [cited 2022 Jun 14];1–12. Available from: https://link.springer.com/article/10.1007/s12144-021-01548-3

27. Larson R, Csikszentmihalyi M. The experience sampling method. In: Flow and the Foundations of Positive Psychology: The Collected Works of Mihaly Csikszentmihalyi [Internet]. Springer Netherlands; 2014 [cited 2022 Dec 9]. p. 21–34. Available from: https://link.springer.com/chapter/10.1007/978-94-017-9088-8_2

28. Raja SN, Carr DB, Cohen M, Finnerup NB, Flor H, Gibson S, et al. The revised International Association for the Study of Pain definition of pain: concepts, challenges, and compromises [Internet]. Vol. 161, Pain. NIH Public Access; 2020 [cited 2022 Jun 14]. p. 1976–82. Available from: /pmc/articles/PMC7680716/

For peer review only

29. NICE. Chronic pain (primary and secondary) in over 16s: assessment of all chronic pain and management of chronic primary pain [Internet]. NICE Guidelines. 2021 [cited 2022 Jun 14]. NICE guideline [NG193]. Available from: https://www.ncbi.nlm.nih.gov/books/NBK569983/bin/niceng193er6_bm1.pdf

30. Tang NKY, Goodchild CE, Sanborn AN, Howard J, Salkovskis PM. Deciphering the temporal link between pain and sleep in a heterogeneous chronic pain patient sample: a multilevel daily process study. Sleep [Internet]. 2012 May 1 [cited 2021 Nov 10];35(5):675–87. Available from: https://pubmed.ncbi.nlm.nih.gov/22547894/

31. Magezi D a. Linear mixed-effects models for within-participant psychology experiments: an introductory tutorial and free, graphical user interface (LMMgui). Front Psychol [Internet]. 2015;6(January):1–7. Available from: http://journal.frontiersin.org/Journal/10.3389/fpsyg.2015.00002/abstract

32. Epskamp S, Borsboom D, Fried EI. Estimating psychological networks and their accuracy: A tutorial paper. Behav Res Methods [Internet]. 2018 Feb 1 [cited 2022 Jun 14];50(1):195–212. Available from: https://link.springer.com/article/10.3758/s13428-017-0862-1

33. Cleeland CS, Ryan KM. Pain assessment: global use of the Brief Pain Inventory. [Internet]. Vol. 23, Annals of the Academy of Medicine, Singapore. 1994 [cited 2022 Jun 14]. p. 129–38. Available from: https://europepmc.org/article/med/8080219

34. Roelofs J, Peters ML, McCracken L, Vlaeyen JWS. The pain vigilance and awareness questionnaire (PVAQ): Further psychometric evaluation in fibromyalgia and other chronic pain syndromes. Pain. 2003 Feb 1;101(3):299–306.

For peer review only

35. Woby SR, Roach NK, Urmston M, Watson PJ. Psychometric properties of the TSK-11: A shortened version of the Tampa Scale for Kinesiophobia. Pain. 2005 Sep 1;117(1–2):137–44.

36. Cane D, Nielson WR, McCarthy M, Mazmanian D. Pain-related activity patterns: Measurement, interrelationships, and associations with psychosocial functioning. Clin J Pain [Internet]. 2013 May [cited 2022 Jun 14];29(5):435–42. Available from: https://journals.lww.com/clinicalpain/Fulltext/2013/05000/Pain_related_Activity_Patterns__Measurement,.8.aspx

37. Sullivan MJL, Bishop SR, Pivik J. The Pain Catastrophizing Scale: Development and Validation. Psychol Assess. 1995;7(4):524–32.

38. Nicholas MK. The pain self-efficacy questionnaire: Taking pain into account. Eur J Pain. 2007 Feb 1;11(2):153–63.

39. Craig CL, Marshall AL, Sjöström M, Bauman AE, Booth ML, Ainsworth BE, et al. International physical activity questionnaire: 12-Country reliability and validity. Med Sci Sports Exerc [Internet]. 2003 [cited 2022 Jun 14];35(8):1381–95. Available from: http://www.acsm-msse.org

40. Syrjala KL, Stover AC, Yi JC, Artherholt SB, Abrams JR. Measuring social activities and social function in long-term cancer survivors who received hematopoietic stem cell transplantation. Psychooncology [Internet]. 2010 May 1 [cited 2022 Jun 27];19(5):462–71. Available from: https://onlinelibrary.wiley.com/doi/full/10.1002/pon.1572

41. Bastien CH, Vallières A, Morin CM. Validation of the insomnia severity index as an outcome measure for insomnia research. Sleep Med. 2001 Jul 1;2(4):297–307.

For peer review only

42. Cohen S, Kamarck T, Mermelstein R. A global measure of perceived stress. J Health Soc Behav. 1983;24(4):385–96.

43. Zigmond AS, Snaith RP. The Hospital Anxiety and Depression Scale. Acta Psychiatr Scand [Internet]. 1983 Jun 1 [cited 2018 Apr 28];67(6):361–70. Available from: http://doi.wiley.com/10.1111/j.1600-0447.1983.tb09716.x

44. Osman A, Bagge CL, Gutierrez PM, Konick LC, Kopper BA, Barrios FX. The suicidal behaviors questionnaire-revised (SBQ-R): Validation with clinical and nonclinical samples. Assessment [Internet]. 2001 Jul 26 [cited 2022 Jun 14];8(4):443–54. Available from: https://journals.sagepub.com/doi/abs/10.1177/107319110100800409

45. Raes F, Pommier E, Neff KD, Van Gucht D. Construction and factorial validation of a short form of the Self-Compassion Scale. Clin Psychol Psychother [Internet]. 2011 May 1 [cited 2022 Jun 14];18(3):250–5. Available from: https://onlinelibrary.wiley.com/doi/full/10.1002/cpp.702

46. Herdman M, Gudex C, Lloyd A, Janssen M, Kind P, Parkin D, et al. Development and preliminary testing of the new five-level version of EQ-5D (EQ-5D-5L). Qual Life Res [Internet]. 2011 Dec 9 [cited 2022 Jun 14];20(10):1727–36. Available from: https://link.springer.com/article/10.1007/s11136-011-9903-x

47. Carney CE, Buysse DJ, Ancoli-Israel S, Edinger JD, Krystal AD, Lichstein KL, et al. The consensus sleep diary: Standardizing prospective sleep self-monitoring [Internet]. Vol. 35, Sleep. Oxford Academic; 2012 [cited 2022 Jun 14]. p. 287–302. Available from: https://academic.oup.com/sleep/article/35/2/287/2558899

48. Ancoli-Israel S. Actigraphy. Princ Pract Sleep Med. 2005;1459–67.

For peer review only

49. Williams JM, Taylor DJ, Slavish DC, Gardner CE, Zimmerman MR, Patel K, et al. Validity of Actigraphy in Young Adults With Insomnia. https://doi.org/101080/1540200220181545653 [Internet]. 2018 Jan 2 [cited 2022 Jun 27];18(1):91–106. Available from: https://www.tandfonline.com/doi/abs/10.1080/15402002.2018.1545653

50. Mccall C, Mccall WV. Comparison of actigraphy with polysomnography and sleep logs in depressed insomniacs. J Sleep Res. 2012 Feb;21(1):122–7.

51. Fekedulegn D, Andrew ME, Shi M, Violanti JM, Knox S, Innes KE. Actigraphy-Based Assessment of Sleep Parameters. Ann Work Expo Heal [Internet]. 2020 Apr 30 [cited 2022 Jun 27];64(4):350–67. Available from: https://academic.oup.com/annweh/article/64/4/350/5735350

52. Ancoli-Israel S, Martin JL, Blackwell T, Buenaver L, Liu L, Meltzer LJ, et al. The SBSM Guide to Actigraphy Monitoring: Clinical and Research Applications. https://doi.org/101080/1540200220151046356 [Internet]. 2015 Jul 17 [cited 2022 Jun 27];13:S4–38. Available from: https://www.tandfonline.com/doi/abs/10.1080/15402002.2015.1046356

53. Benjamini Y, Hochberg Y. Controlling the False Discovery Rate: A Practical and Powerful Approach to Multiple Testing. J R Stat Soc Ser B [Internet]. 1995 Jan 1 [cited 2022 Jun 28];57(1):289–300. Available from: https://onlinelibrary.wiley.com/doi/full/10.1111/j.2517-6161.1995.tb02031.x

54. Akaike H. Information Theory and an Extension of the Maximum Likelihood Principle. In Springer, New York, NY; 1998 [cited 2022 Jun 27]. p. 199–213. Available from: https://link.springer.com/chapter/10.1007/978-1-4612-1694-0_15

55. Akaike H. A New Look at the Statistical Model Identification. IEEE Trans

For peer review only

Automat Contr. 1974;19(6):716–23.

56. Schwarz SG. “Estimating the Dimension of a Model.” Ann Stat [Internet]. 1978 [cited 2022 Jun 27];6(2):461–4. Available from: http://www.citeulike.org/user/abhishekseth/article/90008

57. Bates D, Mächler M, Bolker BM, Walker SC. Fitting linear mixed-effects models using lme4. J Stat Softw [Internet]. 2015 Jun 23 [cited 2022 Jun 27];67(1). Available from: https://arxiv.org/abs/1406.5823v1

58. R Core Team. R: A Language and Environment for Statistical Computing. Vienna R foundation for Statistical Computing.; 2012.

59. Epskamp S, Waldorp LJ, Mõttus R, Borsboom D. The Gaussian Graphical Model in Cross-Sectional and Time-Series Data. Multivariate Behav Res [Internet]. 2018 Jul 4 [cited 2022 Jun 29];53(4):453–80. Available from: https://www.tandfonline.com/doi/abs/10.1080/00273171.2018.1454823

60. Bringmann LF, Vissers N, Wichers M, Geschwind N, Kuppens P, Peeters F, et al. A Network Approach to Psychopathology: New Insights into Clinical Longitudinal Data. PLoS One [Internet]. 2013 Apr 4 [cited 2022 Jun 27];8(4):e60188. Available from: https://journals.plos.org/plosone/article?id=10.1371/journal.pone.0060188

61. Matthews KA, Patel SR, Pantesco EJ, Buysse DJ, Kamarck TW, Lee L, et al. Similarities and differences in estimates of sleep duration by polysomnography, actigraphy, diary, and self-reported habitual sleep in a community sample. Sleep Heal. 2018 Feb 1;4(1):96–103.

12. FIGURE LEGEND

Figure 1

The study uses a prospective experience sampling design involving in vivo data gathering using survey and actigraphy. The participants are asked to engage with the data collection process over 7 consecutive days (8 nights) twice, six-months

For peer review only

apart. An example data collection procedure in a single day is detailed in the box of dashed outline. The participants are prompted to complete a sleep diary in the morning and 3 daily surveys each day. The participants are also asked to wear an actigraph during the entire 7-day (8-night) period for each wave, generating objective estimates of sleep and physical activity. The timing of the diary and surveys is pre-specified. If a participant’s typical wake time is 08:00, a prompt to complete the sleep diary will be sent at 08:30, then the first, second and third daily surveys at 11:00, 13:30 and 16:00.

For peer review only

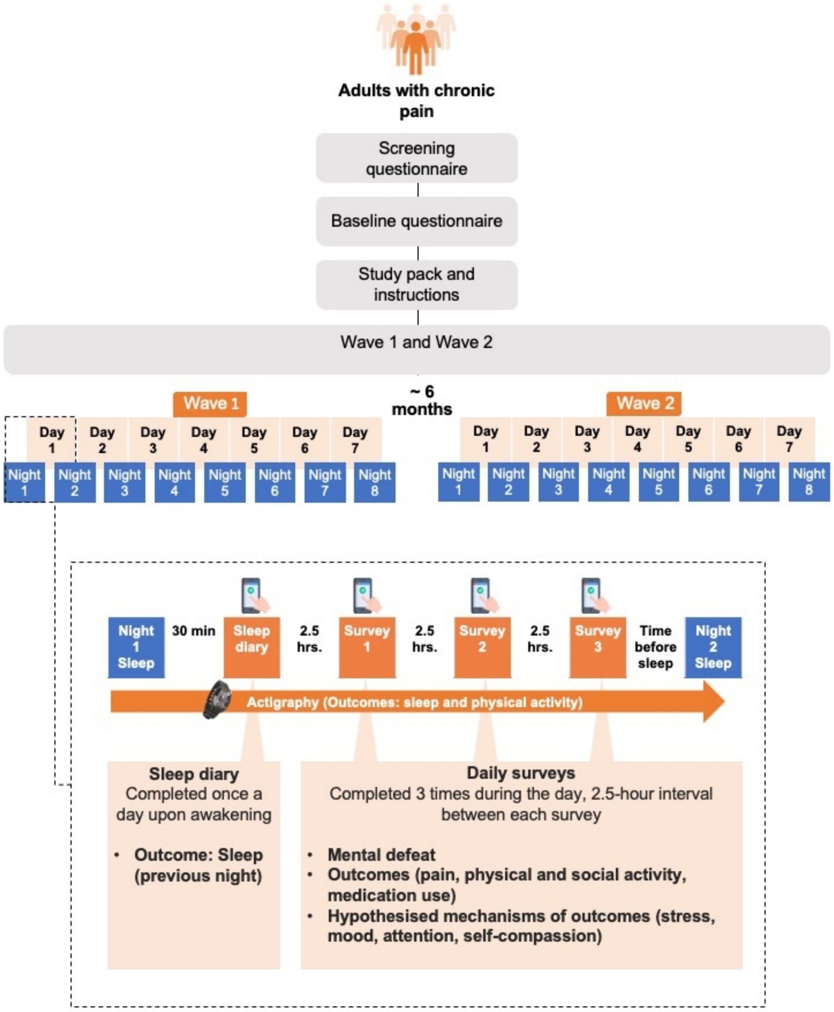

Figure 1. Study design

190x275mm (96 x 96 DPI)

COVID Checklist

The checklist is administered by a member of the research team over the telephone once per person, per timepoint (T1 and T2). This is to ensure reduced risk of virus transmission and to ensure that the sleep, pain, mood and activity measured within the study are representative of their typical chronic pain and not influenced by COVID infection. The COVID checklist requires participants to answer three questions to determine any recent exposure:

1. Have you experienced any of the main COVID-19 symptoms in the last 48 hours?
2. In the last 7 days have you come into close contact with anyone who has tested positive for COVID-19 to your knowledge?
3. Are you or is anyone in your household/bubble currently self-isolating?

If a participant answers 'yes' to one or multiple questions, they will be asked to postpone their participation until they no longer test positive. Data obtained from the checklist will not be analysed as their purposes are for health and safety and to maintain data integrity.

For peer review only
